# Genes related to mitochondrial functions are differentially expressed in phosphine-resistant and -susceptible *Tribolium castaneum*

**DOI:** 10.1186/s12864-015-2121-0

**Published:** 2015-11-18

**Authors:** Brenda Oppert, Raul N. C. Guedes, Michael J. Aikins, Lindsey Perkin, Zhaorigetu Chen, Thomas W. Phillips, Kun Yan Zhu, George P. Opit, Kelly Hoon, Yongming Sun, Gavin Meredith, Kelli Bramlett, Natalie Supunpong Hernandez, Brian Sanderson, Madison W. Taylor, Dalia Dhingra, Brandon Blakey, Marcé Lorenzen, Folukemi Adedipe, Frank Arthur

**Affiliations:** USDA Agricultural Research Service, Center for Grain and Animal Health Research, Manhattan, KS USA; Departamento de Entomologia, Universidade Federal de Vicosa, Vicosa, Brazil; Department of Entomology, Kansas State University, Manhattan, KS USA; Department of Entomology and Plant Pathology, Oklahoma State University, Stillwater, OK USA; LifeTechnologies, Carlsbad, CA USA; Present address: Illumina Inc., San Diego, CA USA; Present address: Nanostring Technologies, Seattle, WA USA; Department of Entomology, North Carolina State University, Raleigh, NC USA

**Keywords:** Anti-diuretic peptide, Cytochrome P450, Deltamethrin, Dihydrolipoamide dehydrogenase, Fumigants, Insecticide resistance, Gene expression, Phosphine resistance, Red flour beetle, RNA-Seq, Stored product pests, *Tribolium castaneum*

## Abstract

**Background:**

Phosphine is a valuable fumigant to control pest populations in stored grains and grain products. However, recent studies indicate a substantial increase in phosphine resistance in stored product pests worldwide.

**Results:**

To understand the molecular bases of phosphine resistance in insects, we used RNA-Seq to compare gene expression in phosphine-resistant and susceptible laboratory populations of the red flour beetle, *Tribolium castaneum*. Each population was evaluated as either phosphine-exposed or no phosphine (untreated controls) in triplicate biological replicates (12 samples total). Pairwise analysis indicated there were eight genes differentially expressed between susceptible and resistant insects not exposed to phosphine (i.e., basal expression) or those exposed to phopshine (>8-fold expression and 90 % C.I.). However, 214 genes were differentially expressed among all four treatment groups at a statistically significant level (ANOVA, *p* < 0.05). Increased expression of 44 cytochrome P450 genes was found in resistant vs. susceptible insects, and phosphine exposure resulted in additional increases of 21 of these genes, five of which were significant among all treatment groups (*p* < 0.05). Expression of two genes encoding anti-diruetic peptide was 2- to 8-fold reduced in phosphine-resistant insects, and when exposed to phosphine, expression was further reduced 36- to 500-fold compared to susceptible. Phosphine-resistant insects also displayed differential expression of cuticle, carbohydrate, protease, transporter, and many mitochondrial genes, among others. Gene ontology terms associated with mitochondrial functions (oxidation biological processes, monooxygenase and catalytic molecular functions, and iron, heme, and tetrapyyrole binding) were enriched in the significantly differentially expressed dataset. Sequence polymorphism was found in transcripts encoding a known phosphine resistance gene, dihydrolipoamide dehydrogenase, in both susceptible and resistant insects. Phosphine-resistant adults also were resistant to knockdown by the pyrethroid deltamethrin, likely due to the increased cytochrome P450 expression.

**Conclusions:**

Overall, genes associated with the mitochondria were differentially expressed in resistant insects, and these differences may contribute to a reduction in overall metabolism and energy production and/or compensation in resistant insects. These data provide the first gene expression data on the response of phosphine-resistant and -susceptible insects to phosphine exposure, and demonstrate that RNA-Seq is a valuable tool to examine differences in insects that respond differentially to environmental stimuli.

**Electronic supplementary material:**

The online version of this article (doi:10.1186/s12864-015-2121-0) contains supplementary material, which is available to authorized users.

## Background

Phosphine is used worldwide as a fumigant to control insect populations in commodities such as stored grains and grain products. Phosphine kills insects rapidly at a relatively low cost, leaving minimal residues on treated products. The continued use of phosphine is of critical importance due to the regulated phase-out of the ozone-depleting fumigant methyl bromide. Phosphine is toxic to all animals, and the Food and Agriculture Organization of the United Nations [1] has established guidelines with recommended times and doses for treatment of stored product pests, depending on environmental conditions. Phosphine is a strong reducing agent, and biological redox systems, particularly the mitochondrial electron transport chain, are likely targets in insects [2]. Phosphine is not absorbed (i.e., not toxic) under anoxic conditions [3], and carbon dioxide can potentiate its toxicity [4], which implies that phosphine targets respiration in insects.

Most bulk grain in the United States is fumigated with phosphine because it is more economical than sampling and monitoring [5]. Therefore, selection pressure for resistance to phosphine is strong in stored product pests. Recent studies indicated a substantial increase in phosphine resistance over the past 21 years in major stored-wheat pests in the United States, with levels of resistance approaching those reported for stored-grain pest species in other countries [6, 7].

The first report of phosphine resistance in the red flour beetle, *Tribolium castaneum*, was more than 40 years ago [8]. In that study, it was demonstrated that 10-fold resistance to phosphine could be selected in six generations from a population of *T. castaneum.* Other studies have suggested that insects (including *T. castaneum*) with low carbon dioxide production display higher resistance to phosphine, suggesting that lower respiration rates reduce fumigant uptake in resistant insects [9]. A major phosphine resistance gene, dihydrolipoamide dehydrogenase (DLD), was found to be mutated in phosphine-resistant *T. castaneum* and another stored product beetle, the lesser grain borer (*Rhyzopertha dominica*), as well as phosphine-resistant nematodes *Caenorhabditis elegans* [10]*.* DLD is a mitochondrial homodimer enzyme and participates in a number of key energy complexes, and thus is highly conserved among organisms.

In this study, we compared a phosphine-resistant and -susceptible population of *T. castaneum*, exposed or not to phosphine, by the differential transcript expression method RNA-Seq, using the sequenced genome of *T. castaneum* to facilitate data analysis. Phosphine-resistant insects also were evaluated for resistance to another insecticide, deltamethrin. We found many statistically-significant differences in gene expression in resistant insects that suggest major changes in the mitochondria. The data indicate that RNA-Seq can be used to identify differential expression patterns in insecticide resistant insects associated with biological mechanisms, as well as individual variation in potential resistance genes within a population.

## Results

### RNA-Seq analysis

To examine differential gene expression associated with phosphine-resistance in *T. castaneum*, we compared expression levels in a phosphine-resistant population from Brazil, reported to have 164-fold resistance to phosphine and a lower respiration rate than susceptible insects [9]. Interestingly, we found that resistance to phosphine in the Brazilian strain had increased nearly 3-fold (to 475-fold) since the previous bioassays were performed (Additional file 1), possibly due to a genetic bottleneck during subculturing for this study. RNA-Seq was used to compare gene expression in this phosphine-resistant strain and a laboratory strain that was susceptible to phosphine, before and after exposure to sublethal phosphine doses/exposure times (for the susceptible insects, the dose was 0.65 ppm for 8 h; for the resistant insects, the dose was 200 ppm for 20 h).

Pairwise analysis was used to construct scatter plots demonstrating the relative expression of genes in untreated susceptible and resistant *T. castaneum* adults (Additional file 2A) and phosphine-treated susceptible and resistant adults (Additional file 2B). Gene expression was also compared in phosphine-treated and untreated resistant insects (Additional file 2C), and phosphine-treated and untreated susceptible insects (Additional file 2D). Plots of relative transcript expression were linear (R^2^ = 0.95 to 0.97), and included genes that were statistically significant in the pairwise analysis (>90 % C.I.), although many were below the cutoff threshold of RPKM < 1 (log_2_). Differences greater than 8-fold were indicated by white dots in the scatter graphs.

In the comparison of resistant to susceptible insects not exposed to phosphine (i.e. basal gene expression, >90 % C.I.), a gene encoding a hypothetical protein was significantly increased in expression (2,630-fold) in resistant compared to susceptible insects (Table 1). Increased expression also was associated with genes encoding cytochrome P450 (CYP) 9e2 (8.84-fold). A gene encoding a glycine-rich protein was decreased in resistant insects 15.6-fold compared to susceptible insects.

When resistant and susceptible insects were exposed to sublethal doses of phosphine, two additional CYP genes (CYP6a14 and 346B-1) were significantly increased in expression in resistant insects compared to susceptible (51.4- and 9.89-fold, respectively; Table 1). Decreased expression in phosphine-exposed resistant insects was found in genes encoding an uncharacterized protein, pox-neuro, and anti-diruetic peptide (ADF; 27.0- to 35.7-fold).

In the comparisons of phosphine-exposed/not exposed for either resistant or susceptible insects, while there were significant differences in gene expression, all RPKM values were less than 1.0 (data not shown).

ANOVA of differences in gene expression among all four treatment groups yielded 214 genes that were significant in the differentially-expressed (DE) dataset (*p* < 0.05; Additional file 3: Table S1). Overall, there were a number of CYP and other genes involved in mitochondrial functions, as well as those related to carbohydrate, protease, and cuticle. In the following paragraphs, we examined more closely the most up- and down-regulated genes and possible correlations to responses to phosphine.

**Table 1:** Results of enrichment analysis (Blast2GO) of GO terms in the dataset of significantly DE genes (Additional file 4: Table S2 and Additional file 5: Table S1)

GO-ID	Term	Category^a^	FDR^b^	P-Value	#Test	#Ref	#notAnnot Test	#notAnnot Ref
GO:0005506	iron ion binding	F	3.99E-07	9.40E-11	17	153	115	9554
GO:0055114	oxidation-reduction process	P	1.09E-06	6.16E-10	31	636	101	9071
GO:0016491	oxidoreductase activity	F	1.09E-06	7.68E-10	31	642	101	9065
GO:0004497	monooxygenase activity	F	6.16E-06	5.87E-09	13	106	119	9601
GO:0020037	heme binding	F	6.16E-06	8.61E-09	15	157	117	9550
GO:0046906	tetrapyrrole binding	F	6.16E-06	9.33E-09	15	158	117	9549
GO:0044710	single-organism metabolic process	P	6.16E-06	1.02E-08	52	1747	80	7960
GO:0016705	oxidoreductase activity, acting on paired donors, with incorporation	F					117	9534
	or reduction of molecular oxygen		1.53E-05	2.88E-08	15	173		
GO:0003824	catalytic activity	F	5.33E-03	1.13E-05	75	3644	57	6063

^a^F: Molecular Function); P: Biological Process

^b^[25]

Unfortunately, the most highly upregulated gene among all groups in the DE dataset was the uncharacterized protein (LOC103314140) found in the pairwise analysis (Additional file 3: Table S1); this gene was 2,600-fold increased in resistant compared to susceptible insects but decreased to 833-fold when exposed to phosphine, and contained a motif similar to DUF753 Transglycosylase (data not shown). A gene encoding an alanine and glycine-rich protein was up-regulated mostly in phosphine-exposed resistant insects; these proteins are found in mammals and plants but their function is unknown. There were twelve significantly up-regulated CYP genes in phosphine-resistant insects, and phosphine exposure resulted in further increases in the expression of nine CYP genes (CYP genes will be discussed in a separate section below). Several genes encoding UGT were significantly up-regulated in resistant insects, although one encoding UGT-2B7 had low RPKM values (data not shown). UGTs serve a major role in the conjugation and subsequent elimination of potentially toxic xenobiotics and endogenous compounds, and UGT-2B7 is the isoform most responsible for glucuronidation of clinical drugs [11]. UGT enzymes are found in a number of metabolic pathways in *T. castaneum*, including cytochrome P450 systems involved in the metabolism of xenobiotics. Some genes encoding enzymes active in mitochondria were increased in expression in resistant insects, and further increased when insects were exposed to phosphine, including one encoding peroxiredoxin-6, which protects against mitochondrial dysfunction in mice [12], and dihydrolipoyllysine-residue succinyltransferase (DLST) and associated enzyme dihydrolipoyl dehydrogenase, another name for the major phosphine resistance gene DLD [10]. Up-regulated genes in resistant insects also were associated with the cuticle, including one encoding a gene similar to keratin-associated protein 19-3-like, which in arthropods promotes immobilization of invading microbes at injury sites [13]. Genes encoding solute transporters were both up- and down-regulated in resistant insects (Additional file 3: Table S1 and Additional file 4: Table S2).

Several severely down-regulated genes in resistant insects encoded ADFs, and while phosphine exposure increased ADF expression in susceptible insects, expression was decreased further in resistant insects, suggesting increased water secretion (Additional file 4: Table S2). Another gene, venom allergen 3, was decreased in resistant insects regardless of phosphine exposure. The down-regulated group also included genes encoding proteins that function in cuticle, sugar transport, and mitochondria. A cathepsin B was downregulated in resistant insects, and this gene is expressed at low levels throughout the life stages of *T. castaneum* (Perkin et al., unpublished) and is likely a lysosomal enzyme that may activate caspase during mitochondrial stress. A gene encoding enolase, an enzyme involved in the penultimate step of glycolysis, was severely down-regulated in resistant insects. There were a number of genes with uncharacterized functions and without chromosome placement in the latest genome assembly (ie, “un” indicating unplaced scaffold) in the down-regulated group.

Enrichment analysis indicated that GO terms associated with metabolism (GO:0044710, Biological Process—BP) were significantly enriched in the DE dataset (FDR = 6.2e^−6^; Table 2 ). Others that suggest association with mitochondrial functions, such as oxidation-reduction process (GO:0055114, BP), and oxidoreductase activity (GO:0016491 and GO:0016705, Molecular Function—MF), were significantly enriched in the DE dataset (FDR ≤ 1.5e^−5^). In conjunction with oxidation/reduction reactions, genes encoding proteins that have iron binding (GO:005506, MF, FDR = 4.0e^−7^), heme binding (GO:0020037, MF, FDR = 6.2e^−6^), and tetrapyrrole binding (GO:0046906, MF, FDR = 6.2e^−6^) properties also were enriched. As noted earlier, CYP genes were included in the enriched monooxygenase activity (GO:0004497, MF, FDR = 6.2e^−6^) category. Genes encoding enzymes were also enriched (catalytic activity, MF, FDR = 5.3e^−3^). All GO terms were over-represented in the dataset, and support the differential expression of genes involved in metabolic and respiration functions among resistant and susceptible insects.

### Dihydrolipoamide Dehydrogenase (DLD) as a resistance gene

**Fig. 1:**
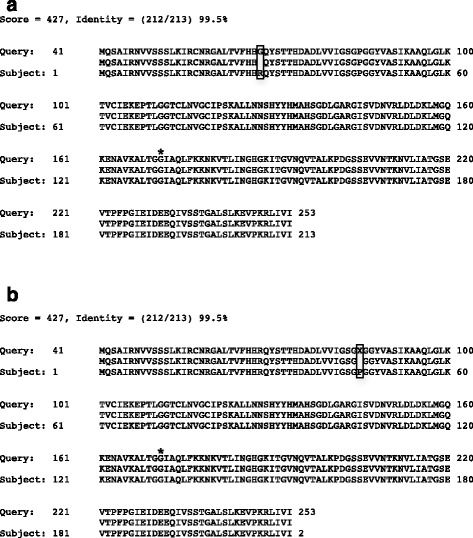
Blastx of putative N-terminal DLD consensus sequences predicted from transcripts from adult *T. castaneum* strains exposed or not to phosphine, with sequences from **a** the phosphine-susceptible strain and **b** phosphine-resistant strain; all were “Queries” to QTC4 “Subject” (gi|399108166|gb|AFP20530.1|). Differences between the phosphine-susceptible, - resistant, and QTC4 sequences are boxed. All letters are standard amino acid abbreviations, except X = unknown (but was a mixture of serine and proline residues). The *asterisk* denotes the G131S phosphine-resistance mutation described in [10]

Since DLD is a phosphine resistance gene in *T. castaneum* [10] and was increased in expression in resistant insects, we used RNASeq data to examine sequences for DLD from phosphine-susceptible and -resistant insects. There was relatively good coverage of reads aligning to DLD from QTC4 (a susceptible *T. castaneum* population, accession JX434604) in all four treatment groups (Additional file 5), although depth of coverage was better in transcripts from the resistant insects in some regions. There have been multiple mutations noted in DLD genes in phosphine-resistant insects and nematodes, but the only mutation that has been identified in a phosphine-resistant population of *T. castaneum* was G131S [10]. However, we did not find this mutation in predicted DLD sequences from susceptible or resistant insects, either exposed or not to phosphine (Fig. 1). We did find a difference in the predicted signal peptide region, R28G, in all sequences from susceptible insects compared to that of *T. castaneum* strain QTC4, whether they were exposed to phosphine or not; this may represent a marker for this population (Fig. 1a). In the phosphine-resistant population, insects unexposed or exposed to phosphine had the polymorphism P45S in the N-terminal FAD binding region of DLD, found in 315/455 (69.2 %) transcripts from the unexposed insects, and 352/546 (64.5 %) transcripts from the phosphine-exposed insects (Fig. 1b). It is possible that this mutation is critical to survival of phosphine exposure, as mutations in this region were also found in other phosphine-resistant insects and nematodes [10]. For the remainder of the alignment, sequences from susceptible-exposed insects were orthologous to that of QTC4 (Additional file 6). SNPs and indels presented uncertainty in alignments of the C-terminal region from transcripts of the susceptible unexposed, and resistant exposed/unexposed (Additional file 7), so we were unable to detail mutations in other regions that may have affected dimerization or NADH binding. We noted a number of indels that resulted in premature stop codons near the beginning of the NADH binding site in transcripts from resistant insects, either exposed or not to phosphine. We also found a few regions in the QTC4 DLD sequence that were different in the DLD transcripts from resistant insects that may be useful in designing a diagnostic assay based on restriction digest analysis for resistance in field populations of *T. castaneum* (shaded sequences, Additional file 6).

### Increased CYP gene expression

**Fig. 2:**
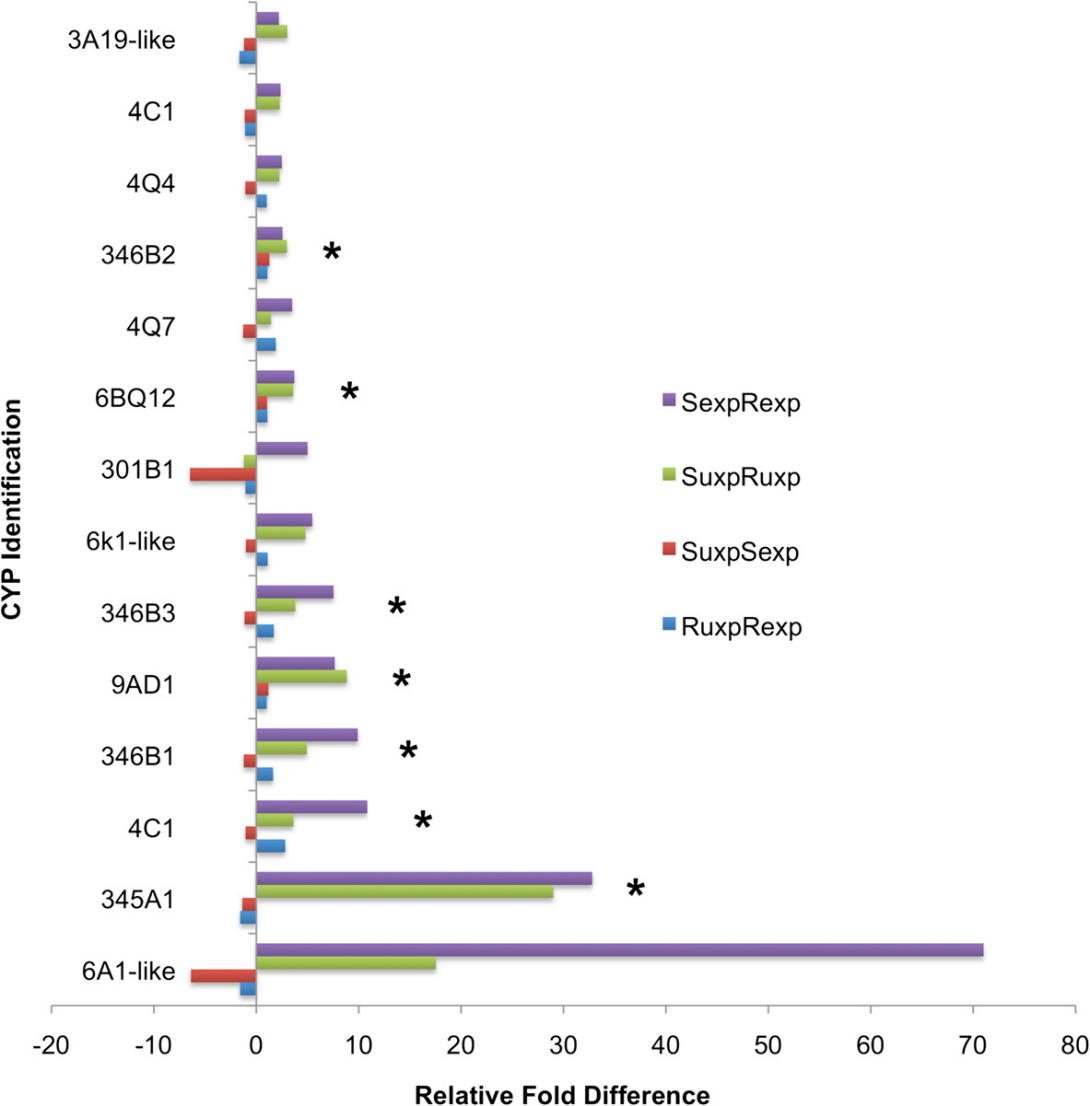
Differential expression of genes (>2-fold) encoding CYPs among all four treatment group comparisons (susceptible vs. resistant phosphine-exposed—SexpRexp; susceptible vs. resistant not exposed—SuxpRuxp; susceptible not exposed vs. phosphine-exposed—SuxpSexp; resistant not exposed vs. phosphine-exposed—RuxpRexp), using F-test (ANOVA, FDR [25]) with those that are significant (*p* < 0.05) marked with an asterisk

CYPs play important roles in the detoxification of xenobiotics, function as monooxygenases, and effectively oxidize organic substrates. Since phosphine is a strong reducing agent, CYPs may act antagonistically in biological systems. CYPs can participate as terminal oxidases in electron transport chains, downstream of DLD in the citric acid cycle. Overall, the expression of 44 genes encoding CYP genes was increased in phosphine-resistant compared to - susceptible *T. castaneum* (data not shown), with 14 differentially expressed more than 2-fold, and five that were significantly increased (*p* < 0.05) under phosphine exposure (Fig. 2). These genes were from clans 3, 4, and mitochondrial, and many were CYP6 genes, located in a gene expansion group on chromosome 4 in *T. castaneum*, and CYP346, located in another gene expansion group on chromosome 5 (data not shown)*.* CYP6 orthologs have been linked to insecticide resistance in *Anopheles gambiae* (mosquito [14, 15]), *Myzus persicae* (aphid [16]), and *D. melanogaster* (fly [17]), among other insects.

### Effect of deltamethrin on different *T. castaneum* populations

**Fig. 3:**
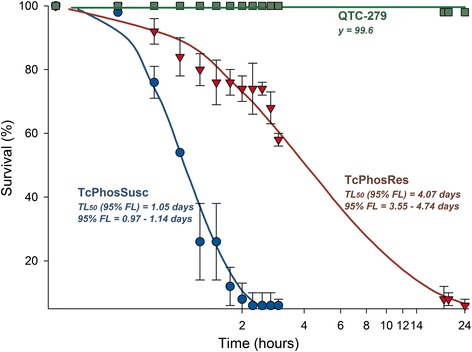
Survival of *T. castaneum* adults exposed to 0.156 AI/cm^2^ deltamethrin on concrete surfaces. *T. castaneum* strains included phosphine-susceptible (TcPhosSusc), phosphine-resistant (TcPhosRes), or QTC-279. The survival curves were significantly different by Cox regression analysis (*χ* ^2^ = 97.23, df = 2, *p* < 0.001) and the median survival time (LT_50_; and respective 95 % fiducial limits) was estimated and indicated for the strains reaching at least 50 % mortality within the exposure period (i.e., 24 hs). The symbols indicate the observed mean values of five replicates (± S.E.)

Resistance to the contact pyrethroid insecticide deltamethrin in *T. castaneum* strain QTC279 was linked to increased CYP expression [18, 19]. Because of the increase in CYP transcripts in the Brazilian phosphine-resistant strain, we predicted that this strain also would be resistant to deltamethrin, especially since this pyrethroid historically has been used heavily in stored grains in Brazil. Therefore, we exposed adults of QTC279 and the phosphine-susceptible and Brazilian phosphine-resistant populations to concrete surfaces treated with deltamethrin at the equivalent of the highest label rate 0.156 mg AI/cm^2^ (Fig. 3). For the first three hours, adults from the Brazilian phosphine-resistant population were able to withstand the pyrethroid exposure, exhibiting a survival profile intermediate between the susceptible strains and the pyrethroid-resistant QTC279. However, after extended exposure (ca. 19 h), the Brazilian insects were knocked-down and immoblized, whereas the QTC279 adults were upright and running in the exposure arena. The susceptible strain was not able to withstand 2 h deltamethrin exposure, reaching 100 % mortality within this period. The deltamethrin formulation currently used in Brazil is synergized with PBO to avoid complications of insecticide resistance by increased CYP expression. The data suggest that deltamethrin exposure may have contributed to the retention of resistance genes in this phosphine-resistant population.

## Discussion

Phosphine resistance threatens the economical control of storage pests worldwide. It is critical to understand the molecular differences in resistant insect populations so that we can develop effective resistance management strategies. This study represents the first to examine phosphine resistance by gene expression analysis. Unfortunately, many insect genes still have undiscovered functions, including some identified in this study as associated with phosphine exposure or resistance. While this limits our interpretation of the results, associations of these genes with phosphine response can help to further delineate their function in future studies.

Regardless, we demonstrated significant changes in the expression of genes in a Brazilian phosphine-resistant population of *T. castaneum*, either exposed or not to phosphine, compared to a phosphine-susceptible population. Most notably, genes encoding CYPs were increased and those encoding anti-diuretics peptides were decreased substantially in the phosphine resistant population, and in most cases the difference was enhanced with phosphine exposure. In addition, many genes associated with mitochondrial functions were differentially expressed, correlated with the likely mode of action of phosphine in insects.

While CYPs are involved in many cases of insecticide resistance, they have not been associated with phosphine resistance. Cyp6BQ9, located on the same linkage group as the most highly upregulated CYP6A1-like in our study (Fig. 2, but also called CYPBQ7, Additional file 3), is expressed in the adult brain of *T. castaneum* and is responsible for deltamethrin resistance in *T. castaneum* strain QTC279 [19]. Phosphine-stimulated expression of CYP genes has not been documented in other insects, and the functional relevance at this point can only be speculated. Increased CYP activity in phosphine environments may be contributing to the detoxification of secondary metabolites due to phosphine exposure (indirect detoxification), or may be a result of uncoupling oxidase and monooxygenase reactions under increased levels of activated oxygen species.

Several genes associated with anti-diuresis functions (ADFs) were down-regulated in phosphine-resistant insects. ADF proteins are anti-diuretic factors involved in osmoregulation; in beetles one ADF is extremely potent at nanomolar concentrations and severely inhibits Malphigian tubule secretion [20]. The data suggest that resistant insects have significantly decreased expression of anti-diuretic peptides compared to susceptible insects, especially when under phosphine stress. Decreased expression of ADFs when resistant insects are exposed to phosphine suggests that insects are decreasing water absorption. In fact, although controversial, forced diuresis has been indicated in mammals in response to complications associated with phosphine intoxication. It is intriguing to speculate that a similar process may be occurring in phosphine-resistant insects.

Based on current information, phosphine likely directly affects the mitochondria and respiration in insects, and genes associated with the mitochondria were both increased and decreased in expression in phosphine-resistant insects. The resistant insect strain in this study has demonstrated a lower respiration rate based on reduced CO_2_ production [7]. Reduced energy metabolism was supported by the fact that the gene encoding enolase, the last enzyme in glycolysis, was severely repressed in resistant insects. The increased expression of UGT, CYPs, and peroxiredoxin-6 in phosphine-exposed resistant insects may be responding to phosphine damage to the mitochondria through the elimination of reactive oxygen species (ROS). The down-regulation of lysosomal cathepsin B in resistant insects may be reducing an apoptotic response to mitochondrial damage through caspases.

DLST binds tightly to DLD in the mitochondria, and both were increased in resistant insects, but slightly decreased in response to phosphine. Examination of transcripts encoding DLD from *T. castaneum* individuals suggested that SNPs and/or indels may contribute to mutations that result in phosphine resistance, with the P45S mutation in the N-terminal FAD binding region likely linked to phosphine resistance in *T. castaneum*. DLD is thus far the only major phosphine resistance gene reported in insects [10], but it is unknown whether mutations can result in increased expression of the gene and/or other genes in resistant insects, such as observed in this study. Good coverage of the DLD sequences from transcripts in all four treatment groups suggested additional SNPs that could be useful in diagnostic assays.

Consistent with the increase in CYP expression in the resistant insects, the Brazilian phosphine-resistant insects were also resistant to deltamethrin. Therefore, caution is warranted in modifying resistance management strategies to control insects in this region. Importantly, these data suggest that *T. castaneum* populations likely have acquired multiple resistance genes in response to field applications of phosphine and pyrethroid insecticide treatments in the past.

## Conclusions

The use of RNA-Seq is an effective tool to evaluate differences in gene expression in different populations. We have demonstrated that the technique provided statistically significant data regarding differences of gene expression in phosphine-resistant and -susceptible populations of *T. castaneum.* The data provides new information on the biological response of insects to phosphine, and offer hints toward the functional relevance of these genes in phosphine resistance. In addition to expression data, we were able to more fully characterize a gene that has been associated with resistance to phosphine in different organisms.

## Methods

### Insect strains

The phosphine-susceptible population of *T. castaneum* for this study was a laboratory colony (CGAHR, Manhattan, KS) used in previous gene expression studies [21, 22]. Exposure of adults to 1.35 ppm phosphine for 72 h at 27 °C resulted in approximately 50 % mortality (LC_50_, or “median lethal concentration”) in a similar wild-type laboratory population [7]. The resistant population was collected in May, 2005, from maize stored in a metallic bin in Bom Despacho County, Minas Gerais (Brazil; 19° 44' 09" S, 45° 15' 07" W) [9]. Probit analysis indicated that the LC_50_ and LC_99_ for phosphine exposure in the Brazilian resistant population are 309 ppm and 775 ppm, respectively (Additional file 1) [23]. The pyrethroid-resistant strain of *T. castaneum*, QTC279, was from the stock *Tribolium* cultures at CGAHR and originated from the state of Queensland in Australia, and was previously characterized as resistant to deltamethrin due to increased cytochrome P450 (CYP) activity [18].

### Insecticide treatments

The outline for phosphine treatment for the RNA-Seq experiment is found in Additional file 8. For phosphine exposure, 50 adults (approximately 2 weeks post emergence) were exposed to sublethal phosphine doses (based on dose response curves, 0.65 ppm for susceptible and 200 ppm for resistant insects) in sealed gas-tight 3.8 L glass jars and were incubated for 8 or 20 h, respectively, at 25 °C. Equivalent groups of susceptible and resistant adults were treated similarly, but without phosphine exposure. There were triplicate biological replicates for each treatment. Insects were flash-frozen in liquid nitrogen and stored at −80 °C in RNA grinding buffer (Qiagen, Valencia, CA, USA).

For deltamethrin treatment, concrete exposure arenas were created by mixing a driveway patching material (Rockkite®, Hartline Products, Co., Inc., Cleveland, OH, USA) with water to create a liquid slurry, and filling the bottom portion of a plastic Petri dish (62 cm^2^ area) to a depth of about 1.25 cm. The arenas were allowed to dry for several days on a laboratory counter. Deltamethrin (Centynal EC®, 50 mg active ingredient [AI]/ml, Central Life Sciences, Shaumberg, IL, USA) was used at the maximum label rate (42.8 ml in 3,784 ml water to cover 94 m^2^). Six individual replicate solutions were formulated in 50 mL volumetric flasks to spray the arenas with the spray rate of 0.3 formulated spray per the 62 cm^2^ area of the arena, which is equivalent to the volumetric spray rate from the label (final deposition of 0.156 AI/cm^2^). An artists' air-brush (Badger Air-Brush Company, Franklin Park, IL, USA) was used to spray each arena. There were five replicates each for the phosphine-susceptible and -resistant populations, and also five replicates of *T. castaneum* QTC279. The arenas were sprayed and allowed to dry overnight, and the next day ten adults from each of the three colonies described above were placed on each arena. The arenas were held on a laboratory counter at room temperature, and beetles were evaluated for knockdown (number of beetles on their dorsal side and unable to flip over) every 15 min for 3 h, and at 19, 20, and 24 h post-exposure. The results obtained were subjected to survival analysis using Cox regression allowing comparison among strains.

### High-throughput sequencing

Total RNA was extracted from treatment groups of adult *T. castaneum* using the RNeasy plus mini kit with on-column DNase treatment (Qiagen, Valencia, CA, USA), and mRNA was extracted from 5 μg total RNA using Dynabeads® mRNA DIRECT™ Micro Kit. RNA integrity of total RNA, mRNA, and cDNA was validated by TapeStation (Agilent Technologies, Santa Clara, CA USA), and quantitation was with a nanophotometer (Implen, Westlake Village, CA USA). Libraries were prepared with the Ion Total RNA-Seq Kit v2 and were individually barcoded (Ion Xpress™ RNA-Seq Barcode 1–16 Kit, Life Technologies, Carlsbad, CA, USA). Templates were prepared (Ion PITM™ Template OT2 200 Kit) and sequenced (Ion PITM™ Sequencing 200 Kit) on Ion Proton™ PITM Chips on the Ion Torrent Personal Genome Machine (Thermo Fisher Scientific, Grand Island, NY). Each chip was run with 4 barcoded samples. The distribution of reads was relatively equal among samples (Additional file 9). Reads were submitted to the NCBI Sequence Read Archive, accession SRP064827.

### Bioinformatic analyses

Data were trimmed by Basecaller (Torrent Suite Software, Thermo Fisher Scientific). Reads were analyzed for differential expression among treatments by mapping reads from each treatment group to Tcas3 (June 6, 2014), using ArrayStar (DNAStar) and normalized by Reads Per Kilobase of template per Million mapped reads (RPKM [24]); additional data on the filtering of reads during the analysis is in Additional file 9. Statistical significance was determined by either pairwise (Student *t*-test) or all groups (F-test ANOVA) using FDR [25]. Restriction enzyme sites were located in QTC4 (gi|399108165|gb|JX434604.1) using NEBcutter2 [26]. Alignments were made with CLUSTAL Omega (ver. 1.2.0 [27]). A custom annotation file for the *T. castaneum* genome was made in Blast2GO (Valencia, Spain), and enrichment analysis was by Fisher’s Exact Test (Blast2GO).

## Additional files

Additional file 1:
**Mortality of adult** ***T. castaneum*** **from Brazil exposed to increasing doses of phosphine (dotted lines represent the 95 % C.I.).** (PDF 350 kb) Additional file 2: Scatter plots of pairwise comparisons demonstrating differential expression of transcripts from phosphine-susceptible or -resistant adults of *T. castaneum* (axes are RPKM as log2). Statistical analysis was a Students t-test, FDR [25]; white dots are transcripts expressed differentially at more than 8-fold (A, B, C) or 4-fold (D), ≥ 90%CI. (PDF 646 kb) Additional file 3:
**Pairwise comparison of significantly (C.I.>90 %) differential expression in transcripts from susceptible unexposed compared to resistant unexposed (SuxpRuxp) and susceptible exposed compared to resistant exposed (SexpRexp), providing relative fold expression for all (RuxpRexp, SuxpSexp, SuxpRuxp, SexpRexp) on the left and RPKM values (log2) on the right.** Bold line separates genes that were significant in the SuxpRuxp (above) and SexpRexp (below) comparisons. (PDF 63 kb) Additional file 3: Table S1.
**Comparison of relative transcript expression among all four treatment groups (resistant exposed – Rexp; resistant unexposed – Ruxp; susceptible exposed – Sexp; susceptible unexposed – Suxp) in which expression was up-regulated in resistant compared to susceptible adults exposed to phosphine, using F-test (ANOVA, p<0.05, P=p values, FDR [** 25 **]), LG=linkage group (chromosome).** Color codes: orange – cytochrome P450; blue – carbohydrate-related; yellow – protease-related; green – mitochondrial; grey – chitin/cuticle-related; beige - solute transporter. Additional file 4: Table S2.
**Comparison of relative transcript expression among all four treatment groups (resistant exposed – Rexp; resistant unexposed – Ruxp; susceptible exposed – Sexp; susceptible unexposed – Suxp) in which expression was down-regulated in resistant compared to susceptible adults exposed to phosphine, using F-test (ANOVA, p<0.05, P=p values, FDR [** 25 **]), LG=linkage group (chromosome).** Color codes: orange – cytochrome P450; blue – carbohydrate-related; yellow – protease-related; green – mitochondrial; grey – chitin/cuticle-related; beige - solute transporter. Additional file 5:
**Relative coverage of transcripts from susceptible (A, B) and phosphine-resistant (C, D)** ***T. castaneum*** **adults from this study, either not exposed (A, C) or exposed (B, D) to phosphine, aligning to DLD mRNA from a phosphine-susceptible** ***T. castaneum*** **adult strain (QTC4, accession JX434604) using SeqMan Pro (DNAStar).** Thin red line – single strand coverage; thick red line – exceeds threshold of coverage; thin green – coverage on both strands; thick green – above threshold coverage; thin cyan – single direction coverage only. (PDF 99 kb) Additional file 6:
**Multiple sequence alignment of RNA-Seq transcriptome data from phosphine-susceptible (TcPhosSusUxp_DLD, TcPhosSusExp_DLD) and -resistant (TcPhosResUxp_DLD, TcPhosResExp_DLD)** ***T. castaneum*** **to DLD mRNA from other strains.** QTC sequences corresponded to gi|399108165|gb|JX434604.1| (QTC4); gi|399108167|gb|JX434605.1| (QTC931); gi|399108169|gb|JX434606.1| (QTC1012); and gi|399108171|gb|JX434607.1| (QTC1389) [10]. R = purine (A or G); M = Amino (A or C); S = Strong (G or C); Y = pyrimidine (C or T); - = deletion; lower case letters indicate polymorphism (most common nucleotide shown). Shaded are recognition sequences for restriction enzymes that may be used to detect resistance, and sites are identified by arrows. (PDF 100 kb) Additional file 7:
**Summary of SNPs/INDELs found in transcripts encoding DLD from phoshine-susceptible (A, unexposed; B, exposed) or -resistant (C, unexposed; D, exposed)** ***T. castaneum*** **adults, when transcripts were assembled to QTC4 ((gi|399108166|gb|AFP20530.1|) using SeqManNGen (DNAStar).** (PDF 115 kb) Additional file 8:
**Treatment and sequencing strategy for** ***T. castaneum*** **adults (RFB).** Abbreviations: RFBSusc, phosphinesusceptible laboratory colony; RFBPhosResBr, phosphine-resistant population from Brazil. Overall there were four treatments: RFBSusc not exposed to phosphine (blue); RFBPhosResBr not exposed to phosphine (orange); RFBSusc and RFBPhos-ResBr exposed to phosphine* (red). Each treatment was in triplicate; lower tier describes the samples that were extracted for mRNA used in the RNA-Seq experiments. (PDF 278 kb) Additional file 9:
**ArrayStar statistics on alignment of reads to Tcas3 (Total Reference bases - 32,060,158; Total Reference sequences - 18,428; Total Genome bases - 160,464,614).** Parameters: mer length = 5; mer repeat threshold = fixed, 150; read assignment qualification - match 80 % of read or 20 bases. (PDF 60 kb)

## Abbreviations

CYP: Cytochrome P450
ADF: Anti-diruetic peptide
DE: Differentially-expressed
DLD: Dihydrolipoamide or dihydrolipoyl dehydrogenase
DLST: Dihydrolipoyllysine-residue succinyltransferase
FAO: Food and Agriculture Organization of the United Nations
ROS: Reactive oxygen species
UGT: UDP-glucuronosyltransferase
