# Supplementary material for: Genes related to mitochondrial functions are differentially expressed in phosphine-resistant and -susceptible Tribolium castaneum
Source: BMC Genomics. 2015 Nov 18;16:968. doi: 10.1186/s12864-015-2121-0 (PMC4650509; doi:10.1186/s12864-015-2121-0)

Additional File 1. Mortality of adult *T. castaneum* from Brazil exposed to increasing doses of phosphine (dotted lines represent the 95% C.I.).

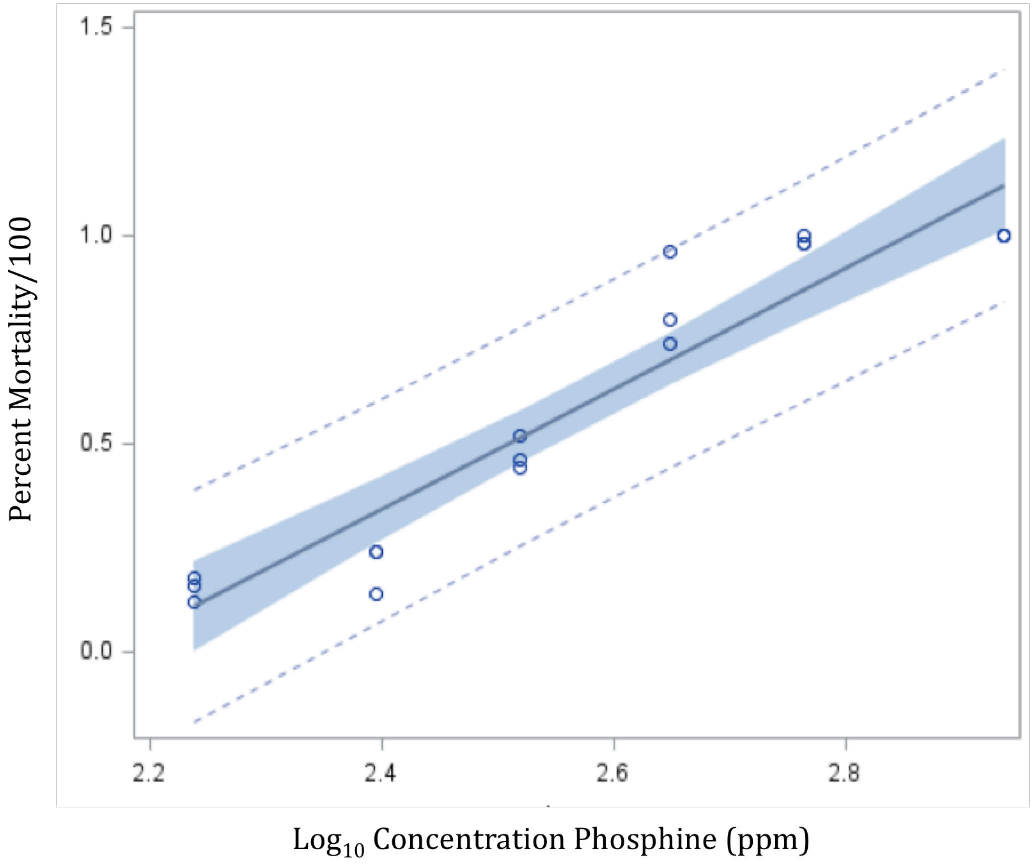

Supplement: Additional file 1: — Mortality of adult T. castaneum from Brazil exposed to increasing doses of phosphine (dotted lines represent the 95 % C.I.). (PDF 350 kb) [file 12864_2015_2121_MOESM1_ESM.pdf]
