# Supplementary material for: Genes related to mitochondrial functions are differentially expressed in phosphine-resistant and -susceptible Tribolium castaneum
Source: BMC Genomics. 2015 Nov 18;16:968. doi: 10.1186/s12864-015-2121-0 (PMC4650509; doi:10.1186/s12864-015-2121-0)
Supplement: Additional file 2: — Scatter plots of pairwise comparisons demonstrating differential expression of transcripts from phosphine-susceptible or -resistant adults of T. castaneum (axes are RPKM as log2). Statistical analysis was a Students t-test, FDR [25]; white dots are transcripts expressed differentially at more than 8-fold (A, B, C) or 4-fold (D), ≥ 90%CI. (PDF 646 kb) [file 12864_2015_2121_MOESM2_ESM.pdf]

Additional File 2. Scatter plots of pairwise comparisons demonstrating differential expression of transcripts from phosphine-susceptible or -resistant adults of *T. castaneum* (axes are RPKM as $\log_2$). Statistical analysis was a Students t-test, FDR [25]; white dots are transcripts expressed differentially at more than 8-fold (A, B, C) or 4-fold (D), ≥ 90% CI.

A) Plot of susceptible (SusUxp) vs. resistant (ResUxp), neither exposed to phosphine, with good linear correlation (R^2 = 0.9532), and 291 genes > 8-fold change (14@ 90%CI, 9 @ 95% CI, and 3 @ 99% CI).

B) Plot of susceptible (SusExp) vs. resistant (ResExp), both exposed to phosphine, good linear correlation (R^2 = 0.9526), and 275 genes > 8-fold change (16 @ 90% CI and 1 @ 99% CI).

C) Plot of ResUxp vs ResExp, good linear correlation (R^2 = 0.9672), and 196 genes > 8-fold change (5 @ 90% CI and 3 @ 99% CI;)

D) Plot of SusUxp vs SusExp, good linear correlation (R^2 = 0.9644), and 217 genes > 8-fold change (7 @ 90% CI and 2 @ 99% CI).

A.

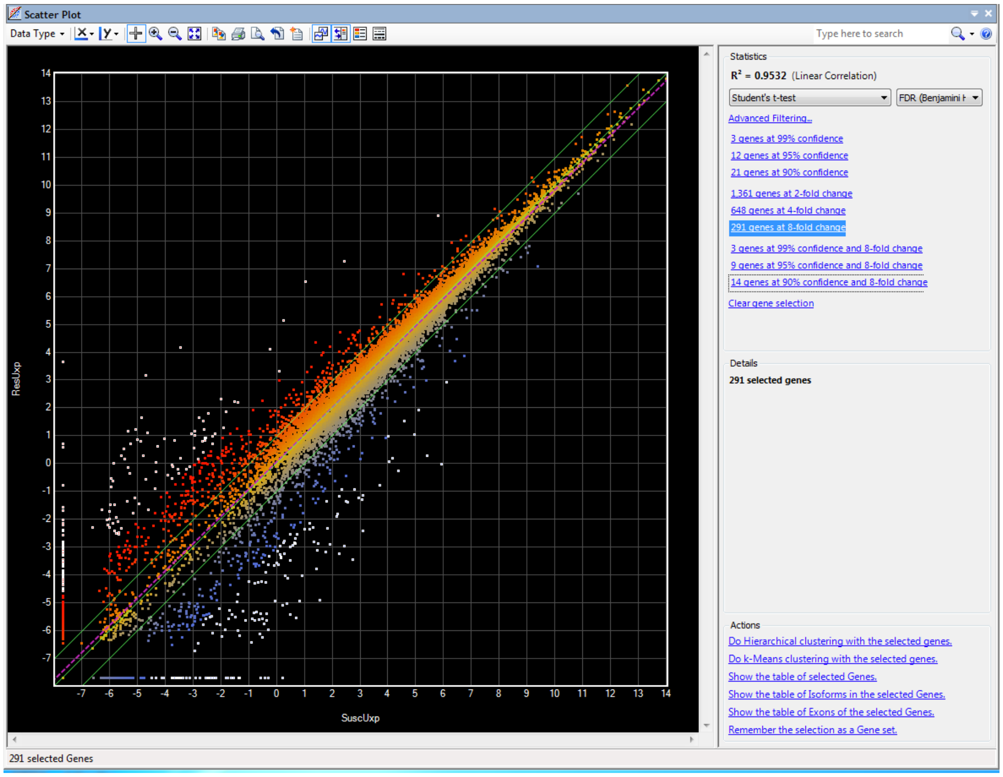
Scatter Plot
Data Type
Type here to search
Statistics
R² = 0.9532 (Linear Correlation)
Student's t-test
FDR (Benjamini H
Advanced Filtering..
3 genes at 99% confidence
12 genes at 95% confidence
21 genes at 90% confidence
1,361 genes at 2-fold change
648 genes at 4-fold change
291 genes at 8-fold change
3 genes at 99% confidence and 8-fold change
9 genes at 95% confidence and 8-fold change
14 genes at 90% confidence and 8-fold change
Clear gene selection
Details
291 selected genes
Actions
Do Hierarchical clustering with the selected genes.
Do k-Means clustering with the selected genes.
Show the table of selected Genes.
Show the table of Isoforms in the selected Genes.
Show the table of Exons of the selected Genes.
Remember the selection as a Gene set.
ResUxp
SuscUxp
291 selected Genes

B.

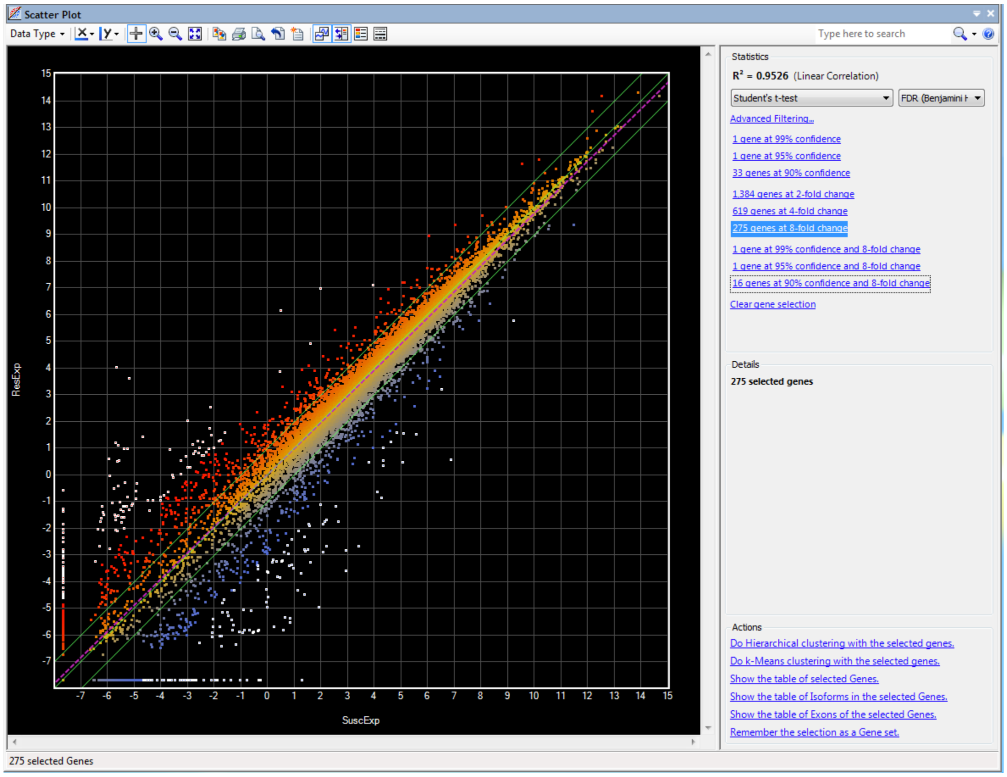
Scatter Plot
Data Type
Type here to search
Statistics
R² = 0.9526 (Linear Correlation)
Student's t-test
FDR (Benjamini H
Advanced Filtering..
1 gene at 99% confidence
1 gene at 95% confidence
33 genes at 90% confidence
1,384 genes at 2-fold change
619 genes at 4-fold change
275 genes at 8-fold change
1 gene at 99% confidence and 8-fold change
1 gene at 95% confidence and 8-fold change
16 genes at 90% confidence and 8-fold change
Clear gene selection
Details
275 selected genes
Actions
Do Hierarchical clustering with the selected genes.
Do k-Means clustering with the selected genes.
Show the table of selected Genes.
Show the table of Isoforms in the selected Genes.
Show the table of Exons of the selected Genes.
Remember the selection as a Gene set.
ResExp
SuscExp
275 selected Genes

C.

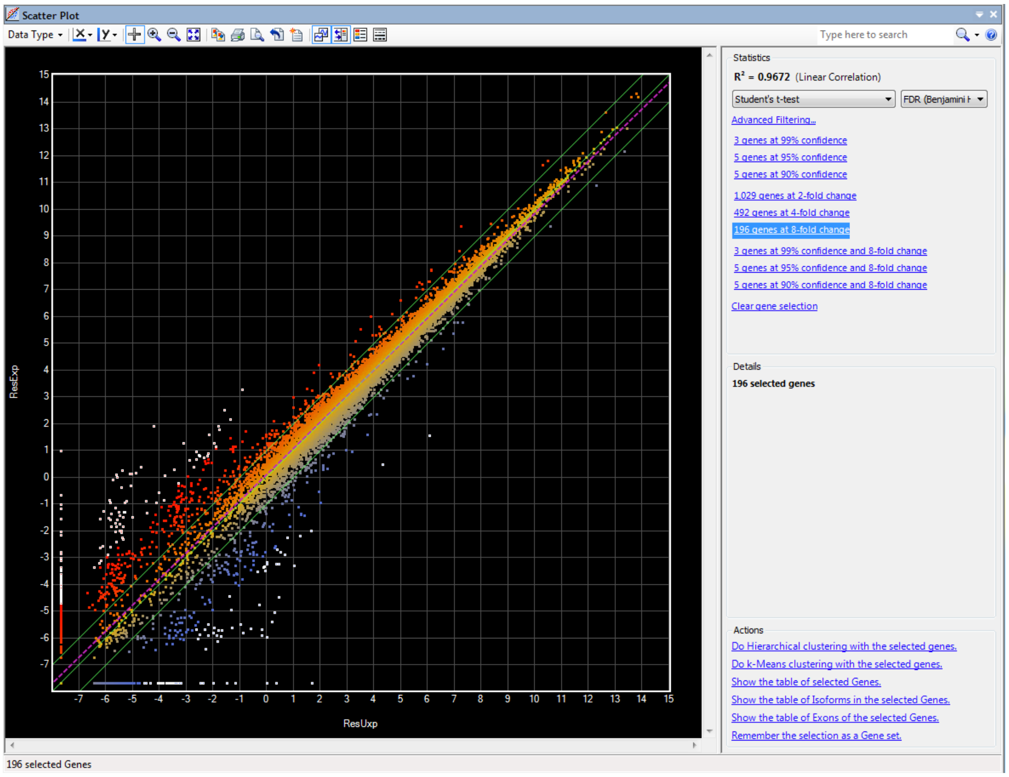

Scatter Plot
Data Type
Type here to search
Statistics
R² = 0.9672 (Linear Correlation)
Student's t-test
FDR (Benjamini H
Advanced Filtering...
3 genes at 99% confidence
5 genes at 95% confidence
5 genes at 90% confidence
1,029 genes at 2-fold change
492 genes at 4-fold change
196 genes at 8-fold change
3 genes at 99% confidence and 8-fold change
5 genes at 95% confidence and 8-fold change
5 genes at 90% confidence and 8-fold change
Clear gene selection
Details
196 selected genes
Actions
Do Hierarchical clustering with the selected genes.
Do k-Means clustering with the selected genes.
Show the table of selected Genes.
Show the table of Isoforms in the selected Genes.
Show the table of Exons of the selected Genes.
Remember the selection as a Gene set.
ResExp
ResUxp
196 selected Genes

D.

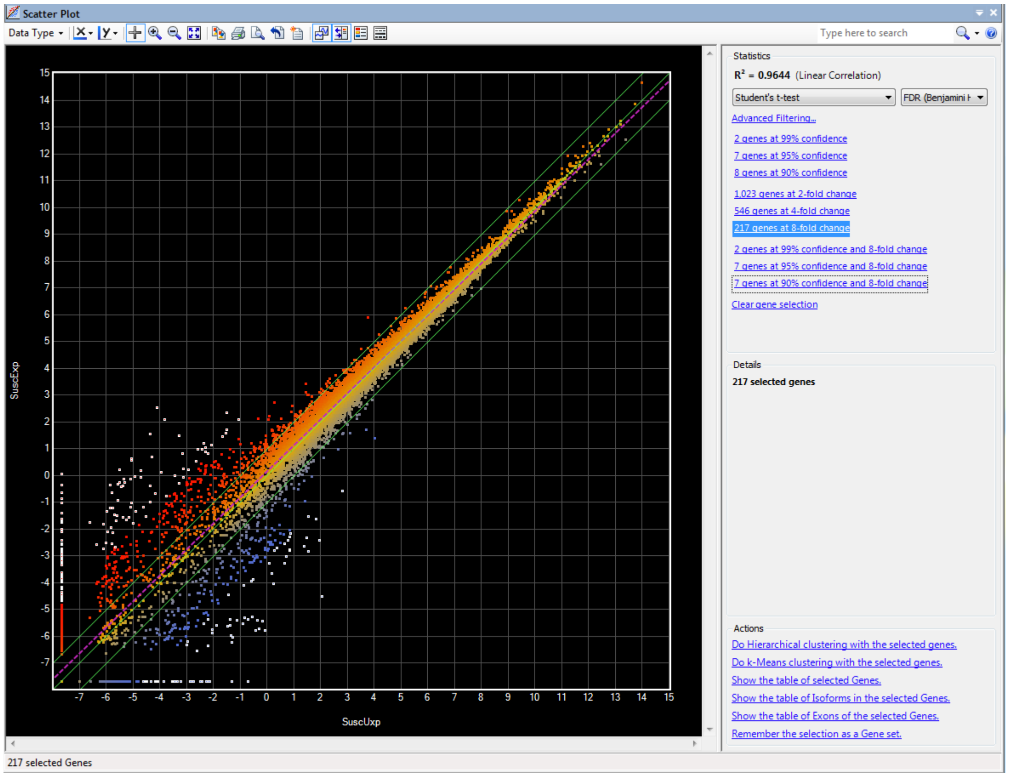

Scatter Plot
Data Type
Type here to search
Statistics
R² = 0.9644 (Linear Correlation)
Student's t-test
FDR (Benjamini H
Advanced Filtering...
2 genes at 99% confidence
7 genes at 95% confidence
8 genes at 90% confidence
1,023 genes at 2-fold change
546 genes at 4-fold change
217 genes at 8-fold change
2 genes at 99% confidence and 8-fold change
7 genes at 95% confidence and 8-fold change
7 genes at 90% confidence and 8-fold change
Clear gene selection
Details
217 selected genes
Actions
Do Hierarchical clustering with the selected genes.
Do k-Means clustering with the selected genes.
Show the table of selected Genes.
Show the table of Isoforms in the selected Genes.
Show the table of Exons of the selected Genes.
Remember the selection as a Gene set.
SuscExp
SuscUxp
217 selected Genes
