# Supplementary material for: Genes related to mitochondrial functions are differentially expressed in phosphine-resistant and -susceptible Tribolium castaneum
Source: BMC Genomics. 2015 Nov 18;16:968. doi: 10.1186/s12864-015-2121-0 (PMC4650509; doi:10.1186/s12864-015-2121-0)
Supplement: Additional file 3: — Pairwise comparison of significantly (C.I.>90 %) differential expression in transcripts from susceptible unexposed compared to resistant unexposed (SuxpRuxp) and susceptible exposed compared to resistant exposed (SexpRexp), providing relative fold expression for all (RuxpRexp, SuxpSexp, SuxpRuxp, SexpRexp) on the left and RPKM values (log2) on the right. Bold line separates genes that were significant in the SuxpRuxp (above) and SexpRexp (below) comparisons. (PDF 63 kb) [file 12864_2015_2121_MOESM3_ESM.pdf]

Additional File 3. Pairwise comparison of significantly (C.I.>90%) differential expression in transcripts from susceptible unexposed compared to resistant unexposed (SuxpRuxp) and susceptible exposed compared to resistant exposed (SexpRexp), providing relative fold expression for all (RuxpRexp, SuxpSexp, SuxpRuxp, SexpRexp) on the left and RPKM values (log_2) on the right. Bold line separates genes that were significant in the SuxpRuxp (above) and SexpRexp (below) comparisons.

Gene	Predicted Function	RuxpRexp	SuxpSexp	SuxpRuxp	SexpRexp	P value	ResExp RPKM	ResUxp RPKM	SuscExp RPKM	SuscUxp RPKM
LOC103314140	hypothetical protein	1.282	4.046	2628.113	833.086	0.001	4.016	3.657	-5.686	-7.702
LOC662432	cyt P450 9AD1	1.026	1.183	8.838	7.659	0.000	8.961	8.925	6.024	5.781
LOC655196	glycine-rich protein	1.224	0.772	0.064	0.102	0.001	0.466	0.174	3.759	4.132
LOC664471	cyt P450 6a14	0.772	0.685	45.620	51.415	0.001	6.162	6.534	0.478	1.023
LOC657187	cyt P450 346B1	1.639	0.816	4.928	9.899	0.003	4.899	4.186	1.591	1.885
LOC103314240	uncharacterized	0.611	1.061	0.064	0.037	0.010	-0.658	0.053	4.096	4.010
LOC661804	pox neuro	1.726	0.697	0.014	0.034	0.086	-2.673	-3.460	2.189	2.711
LOC103313692	ADFb like protein	0.288	1.281	0.127	0.028	0.001	-0.870	0.927	4.262	3.906
