# Supplementary material for: Genes related to mitochondrial functions are differentially expressed in phosphine-resistant and -susceptible Tribolium castaneum
Source: BMC Genomics. 2015 Nov 18;16:968. doi: 10.1186/s12864-015-2121-0 (PMC4650509; doi:10.1186/s12864-015-2121-0)
Supplement: Additional file 3: Table S1. — Comparison of relative transcript expression among all four treatment groups (resistant exposed – Rexp; resistant unexposed – Ruxp; susceptible exposed – Sexp; susceptible unexposed – Suxp) in which expression was up-regulated in resistant compared to susceptible adults exposed to phosphine, using F-test (ANOVA, p<0.05, P=p values, FDR [ 25 ]), LG=linkage group (chromosome). Color codes: orange – cytochrome P450; blue – carbohydrate-related; yellow – protease-related; green – mitochondrial; grey – chitin/cuticle-related; beige - solute transporter. [file 12864_2015_2121_MOESM4_ESM.pdf]

Table 2. Comparison of relative transcript expression among all four treatment groups (resistant exposed – Rexp; resistant unexposed – Ruxp; susceptible exposed – Sexp; susceptible unexposed – Suxp) in which expression was down-regulated in resistant compared to susceptible adults exposed to phosphine, using F-test (ANOVA, $p<0.05$, P=p values, FDR [25]), LG=linkage group (chromosome).

Color codes: orange – cytochrome P450; blue – carbohydrate-related; yellow – protease-related; green – mitochondrial; grey – chitin/cuticle-related; beige - solute transporter.

Gene	Predicted Function	ResExp RPKM	ResUxp RPKM	SuscExp RPKM	SuscUxp RPKM	P	LG
LOC655485	laccase-like	1.250	1.321	1.325	0.355	0.024	un
LOC103312930	slit homolog 3 protein-like	4.415	3.621	4.526	4.176	0.015	5
Lgl	lethal(2) giant larvae protein homolog 1	5.783	5.465	5.919	5.833	0.025	8
LOC658236	uncharacterized	6.149	5.600	6.325	6.096	0.018	X
LOC657938	venom carboxylesterase-6	1.938	1.213	2.157	2.333	0.024	5
LOC658393	inhibitor of nuclear factor kappa-B kinase subunit beta-like	7.401	7.472	7.621	7.741	0.012	un
LOC103313498	protein CTLA-2-alpha-like	6.473	5.950	6.710	6.685	0.011	7
LOC660808	toll-like receptor 8	1.582	1.326	1.867	2.152	0.048	2
LOC660660	DNA-directed RNA polymerases I and III subunit RPAC1	3.660	3.553	3.960	3.053	0.020	7
LOC654849	anamorsin homolog	6.888	6.760	7.196	7.210	0.023	2
LOC663402	membrane metallo-endopeptidase-like 1	3.544	3.476	3.861	3.808	0.023	5
LOC103312937	sodium/potassium-transporting ATPase subunit beta-2-like	2.948	2.946	3.274	2.345	0.008	5
LOC100141891	glycine-rich RNA-binding protein blt801-like	8.254	7.988	8.591	8.587	0.021	5
LOC103312462	kielin/chordin-like protein	4.660	4.716	5.002	5.036	0.031	3
LOC655857	glycoprotein 3-alpha-L-fucosyltransferase A	5.762	6.039	6.107	6.043	0.004	5
LOC103314444	serine--tRNA ligase, mitochondrial	4.590	4.310	4.948	4.836	0.046	10
LOC100142163	proclotting enzyme-like	4.495	4.238	4.870	4.820	0.006	2
LOC656375	Serpin peptidase inhibitor 27	6.040	5.957	6.432	6.174	0.037	9
LOC100141764	uncharacterized	3.925	3.698	4.323	4.262	0.021	7
LOC659625	protein trapped in endoderm-1	3.420	2.801	3.819	3.411	0.003	X
LOC662130	alpha-1,3-mannosyl-glycoprotein 2-beta-N-acetylglucosaminyltransferase	4.641	4.502	5.045	4.926	0.023	7
LOC655360	fibulin-2-like	4.811	4.566	5.225	5.067	0.041	X

LOC664126	protein lifeguard 1	6.683	6.677	7.099	7.027	0.004	3
LOC103314199	uncharacterized	6.501	6.734	6.922	7.049	0.031	9
LOC662467	prostaglandin E synthase 2	5.179	5.092	5.602	5.619	0.036	3
LOC655075	activating signal cointegrator 1 complex	3.821	3.543	4.248	3.961	0.032	8
LOC103313878	DNA ligase 1-like	3.349	2.995	3.785	3.549	0.018	7
LOC103315212	uncharacterized serine-rich protein C215.13	2.986	3.041	3.426	3.799	0.045	2
LOC656166	leucine-rich repeat-containing protein ODA7	3.631	3.518	4.098	3.300	0.032	2
LOC659823	pescadillo homolog	4.936	5.175	5.428	5.431	0.026	un
LOC660945	uncharacterized	1.613	0.398	2.109	2.035	0.046	8
LOC664462	protein fem-1 homolog B	4.474	4.891	5.017	5.075	0.022	7
LOC656699	RINT1-like protein	2.057	1.969	2.605	2.313	0.010	8
LOC661049	beta-lactamase-like protein 2 homolog	8.807	8.674	9.357	9.257	0.012	8
LOC654881	carbohydrate sulfotransferase 11	5.162	5.141	5.713	5.738	0.035	7
LOC663157	uncharacterized	3.959	3.787	4.521	4.159	0.001	7
LOC656168	protein halfway isoform X1	4.574	4.582	5.146	5.201	0.001	3
LOC663631	NFX1-type zinc finger-containing protein 1-like	3.439	3.177	4.023	3.775	0.037	3
Chs2	chitin synthase 2	4.337	4.396	4.938	4.836	0.022	9
LOC655827	cation-independent mannose-6-phosphate receptor	4.576	4.512	5.195	5.087	0.044	X
LOC656743	aquaporin AQPAn.G	4.334	3.856	4.957	4.676	0.044	2
Ago-2b	Argonaute-2b	5.967	5.854	6.609	6.436	0.022	5
LOC658584	glutamate synthase 1 [NADH], chloroplastic	6.278	6.350	6.934	6.958	0.018	5
LOC657806	M-phase phosphoprotein 6	3.592	3.977	4.264	4.565	0.041	5
LOC656292	regulator of G-protein signaling 7	5.107	5.151	5.788	6.031	0.022	9
LOC659272	aldose 1-epimerase-like	6.342	5.995	7.028	6.682	0.028	X
LOC103313065	aromatic-L-amino-acid decarboxylase-like	7.409	7.720	8.117	8.213	0.041	5
LOC658130	probable cytochrome P450 6BK13	5.018	4.827	5.756	5.532	0.001	6
LOC663793	alpha-tocopherol transfer protein	4.541	4.274	5.298	5.209	0.031	9
LOC663781	NFU1 iron-sulfur cluster scaffold homolog, mitochondrial	4.673	4.584	5.433	5.237	0.034	8
LOC659811	sterile alpha and TIR motif-containing protein 1	6.027	6.240	6.797	6.974	0.000	9
LOC655140	retinol dehydrogenase 12-like	2.470	2.305	3.252	3.450	0.018	7
LOC662753	sphingosine-1-phosphate lyase	5.777	5.852	6.606	6.384	0.000	2
LOC656967	NADPH-dependent diflavin oxidoreductase 1	4.848	4.982	5.719	5.679	0.001	9
LOC657319	protein downstream neighbor of son homolog	2.546	2.803	3.419	3.358	0.032	2
LOC659332	guanine nucleotide-binding protein G(f)	3.680	3.525	4.563	4.324	0.044	7
LOC656088	39S ribosomal protein L38, mitochondrial	3.104	3.011	3.989	3.718	0.033	3
LOC103314418	hypothetical protein	2.968	2.521	3.857	3.773	0.017	10

LOC657362	inactive pancreatic lipase-related protein 1	5.106	5.573	6.015	5.947	0.024	9
Rpl41	ribosomal protein L41	8.417	7.818	9.329	9.070	0.042	5
LOC100141680	DNA ligase 3	2.287	2.469	3.212	3.079	0.009	2
LOC661460	2',5'-phosphodiesterase 12	3.884	3.813	4.810	4.590	0.001	5
LOC100142040	similar to odorant binding protein	2.534	2.755	3.521	2.259	0.036	6
LOC656826	similar to pancreatic lipase-related protein 3-like	3.870	3.809	4.865	4.672	0.007	10
LOC663312	UPF0553 protein C9orf64 homolog	5.317	4.953	6.338	6.019	0.004	5
LOC655799	pyruvate dehydrogenase phosphatase regulatory subunit, mitochondrial-like	0.609	0.924	1.646	1.773	0.003	X
LOC662707	leukocyte elastase inhibitor-like	4.627	4.860	5.700	5.872	0.002	5
LOC657080	venom carboxylesterase-6-like	5.101	5.256	6.178	5.910	0.033	10
LOC103315110	protein spaetzle-like isoform X1	1.198	1.442	2.287	2.213	0.040	2
LOC655835	beta-galactosidase-1-like protein 2	2.251	2.079	3.369	2.988	0.045	2
LOC103314086	uncharacterized	8.073	7.563	9.243	9.006	0.008	8
LOC656701	activated CDC42 kinase 1	1.401	1.805	2.581	2.432	0.015	9
LOC656073	chondroitin sulfate synthase 1	3.919	4.388	5.106	4.969	0.006	10
LOC103313602	uncharacterized	2.661	2.837	3.872	3.617	0.040	7
LOC103312224	fibrinogen C domain-containing protein 1-like	3.371	2.827	4.641	4.400	0.006	3
LOC103313145	uncharacterized	4.961	4.886	6.241	6.255	0.036	3
LOC656782	putative inorganic phosphate cotransporter	4.176	4.554	5.459	5.180	0.030	8
LOC655533	phenoloxidase subunit A3	5.648	5.544	6.957	6.774	0.007	6
LOC103312569	uncharacterized	2.040	2.262	3.426	3.367	0.021	4
LOC660240	scavenger receptor class B member 1-like	3.174	2.742	4.566	4.899	0.005	6
LOC100141690	nose resistant to fluoxetine protein 6-like	2.808	3.163	4.210	3.875	0.037	10
LOC660291	spondin-1-like	6.306	7.100	7.726	7.537	0.034	4
LOC103312778	gem-associated protein 8-like	1.419	0.929	2.868	3.425	0.003	4
LOC661067	cytochrome P450 9e2-like	0.485	0.172	1.968	2.168	0.017	8
LOC103314666	nucleic-acid-binding protein from mobile element jockey-like	1.149	1.614	2.646	3.472	0.024	un
Cyp4bn11	cytochrome P450 CYP4BN11	0.721	1.461	2.284	2.457	0.026	5
LOC664159	enolase	5.542	5.517	7.166	7.001	0.011	9
LOC663534	putative fatty acyl-CoA reductase CG5065	2.322	3.025	3.953	3.770	0.041	6
LOC662609	uncharacterized	-0.679	-0.790	0.966	0.173	0.021	6
LOC103314145	uncharacterized	3.139	2.549	4.820	4.596	0.006	3
LOC103313928	uncharacterized	1.282	2.091	2.996	3.090	0.024	8
LOC664297	elongation of very long chain fatty acids protein 1-like	2.136	3.252	3.929	3.758	0.035	3
LOC100142320	2-oxo-4-hydroxy-4-carboxy-5-ureidoimidazoline decarboxylase	5.468	6.154	7.284	7.190	0.014	4

LOC655042	glucose dehydrogenase [FAD, quinone]-like	1.601	0.365	3.525	3.061	0.020	3
LOC661721	hypothetical protein	2.798	2.948	4.750	4.188	0.001	5
LOC103313666	hypothetical protein	0.607	-0.450	2.589	2.481	0.002	7
LOC103314947	uncharacterized	-0.835	-1.528	1.207	0.830	0.014	un
LOC103313167	hypothetical protein	9.353	10.572	11.462	10.968	0.020	5
LOC103312940	hypothetical protein	1.455	1.645	3.589	2.942	0.043	5
LOC103313875	cytochrome b5-related protein-like	4.295	4.464	6.433	6.452	0.001	8
LOC663717	protein regulator of cytokinesis 1	2.922	3.408	5.102	5.195	0.000	7
LOC103312354	mucin-17-like	1.129	-1.295	3.344	3.039	0.037	un
LOC103312942	uncharacterized	3.526	2.915	6.035	6.077	0.001	7
LOC103314246	RNA-directed DNA polymerase from mobile element jockey-like	-1.237	-0.824	1.278	0.683	0.047	un
LOC103315180	receptor-type tyrosine-protein phosphatase C-like	-1.175	-1.857	1.493	0.194	0.013	un
LOC103314834	hypothetical protein	0.401	1.008	3.166	2.636	0.028	un
LOC655077	cathepsin B	1.907	1.529	4.796	4.580	0.001	8
LOC659189	putative inorganic phosphate cotransporter	1.645	2.225	4.560	4.243	0.004	6
LOC659861	uncharacterized	2.558	3.458	5.478	5.269	0.003	6
LOC659613	lipase member I	1.576	3.747	4.820	4.644	0.024	un
LOC655196	glycine-rich protein	0.466	0.174	3.759	4.132	0.001	4
LOC662499	larval cuticle protein A3A	1.002	-0.258	4.304	4.362	0.009	2
LOC658435	cytosolic 10-formyltetrahydrofolate dehydrogenase	1.531	6.055	5.084	4.938	0.015	6
LOC103314477	uncharacterized	-2.334	-1.367	1.355	2.461	0.019	7
LOC664382	keratin, type I cytoskeletal 9	0.536	1.807	4.308	3.724	0.024	2
LOC103313998	uncharacterized protein	-1.700	-2.147	2.185	1.169	0.004	7
LOC103313997	uncharacterized protein	-1.700	-2.147	2.185	1.169	0.004	7
LOC103312356	NFU1 iron-sulfur cluster scaffold homolog, mitochondrial-like	1.513	0.275	5.547	5.371	0.002	un
LOC103314473	uncharacterized	-1.743	-1.151	2.620	3.084	0.005	un
LOC103314240	uncharacterized	-0.658	0.053	4.096	4.010	0.010	un
LOC103314481	uncharacterized	-2.891	-3.184	2.053	1.752	0.003	un
LOC655601	uncharacterized	-6.366	-7.702	-1.231	-1.564	0.007	5
LOC103313692	ADFb like protein	-0.870	0.927	4.262	3.906	0.001	7
LOC659503	venom allergen 3	0.568	-0.012	6.845	5.899	0.003	2
LOC100141528	anti-diruetic peptide precursor	-7.702	0.001	1.271	0.220	0.000	7
