# Supplementary material for: Genes related to mitochondrial functions are differentially expressed in phosphine-resistant and -susceptible Tribolium castaneum
Source: BMC Genomics. 2015 Nov 18;16:968. doi: 10.1186/s12864-015-2121-0 (PMC4650509; doi:10.1186/s12864-015-2121-0)
Supplement: Additional file 4: Table S2. — Comparison of relative transcript expression among all four treatment groups (resistant exposed – Rexp; resistant unexposed – Ruxp; susceptible exposed – Sexp; susceptible unexposed – Suxp) in which expression was down-regulated in resistant compared to susceptible adults exposed to phosphine, using F-test (ANOVA, p<0.05, P=p values, FDR [ 25 ]), LG=linkage group (chromosome). Color codes: orange – cytochrome P450; blue – carbohydrate-related; yellow – protease-related; green – mitochondrial; grey – chitin/cuticle-related; beige - solute transporter. [file 12864_2015_2121_MOESM5_ESM.pdf]

Table 1. Comparison of relative transcript expression among all four treatment groups (resistant exposed – Rexp; resistant unexposed – Ruxp; susceptible exposed – Sexp; susceptible unexposed – Suxp) in which expression was up-regulated in resistant compared to susceptible adults exposed to phosphine, using F-test (ANOVA, $p<0.05$, P=p values, FDR [25]), LG=linkage group (chromosome). Color codes: orange – cytochrome P450; blue – carbohydrate-related; yellow – protease-related; green – mitochondrial; grey – chitin/cuticle-related; beige - solute transporter.

Gene	Predicted Function	ResExp RPKM	ResUxp RPKM	SuscExp RPKM	SuscUxp RPKM	P	LG
LOC103314140	uncharacterized	4.016	3.657	-5.686	-7.702	0.001	3
LOC100141953	UDP-glucuronosyltransferase 2B7-like	0.589	-0.777	-5.989	-7.702	0.007	9
LOC103313334	alanine and glycine-rich protein-like	0.979	-7.702	-5.403	-7.702	0.035	6
CYP6BQ7	probable cytochrome P450 6a14	6.162	6.534	0.478	1.023	0.001	4
LOC659136	cytochrome P450 345A1	6.994	7.269	1.958	2.411	0.000	un
LOC660270	cytochrome P450-like protein	3.858	2.357	0.420	0.497	0.047	5
LOC657187	cytochrome P450 346B1	4.899	4.186	1.591	1.885	0.003	5
OBP-C10	B1 protein-like (olfactory receptor)	7.121	5.588	3.940	4.344	0.039	10
LOC662432	cytochrome P450 9AD1	8.961	8.925	6.024	5.781	0.000	2
LOC657336	cytochrome P450 346B3	5.413	4.633	2.499	2.702	0.017	5
LOC660851	15-hydroxyprostaglandin dehydrogenase [NAD(+)]-like	3.919	3.286	1.255	1.060	0.001	3
LOC103312644	uncharacterized	4.080	4.346	1.508	1.381	0.018	6
LOC662655	solute carrier family 25 member 38-A	9.366	7.231	7.015	7.617	0.001	8
LOC103314496	hypothetical protein	1.766	2.009	-0.528	0.131	0.017	un
LOC658613	glycine dehydrogenase (decarboxylating), mitochondrial	5.506	3.470	3.254	3.603	0.010	3
LOC103315019	cuticle protein CP14.6-like	3.102	3.464	0.874	1.224	0.004	2
LOC659881	regulating synaptic membrane exocytosis protein 2	1.491	1.896	-0.649	-0.078	0.020	3
LOC658401	uncharacterized	11.639	10.283	9.505	9.165	0.047	8
LOC103314100	keratin-associated protein 19-3-like	4.301	3.270	2.239	2.453	0.023	8
LOC660892	yellow-h	2.683	2.342	0.677	0.322	0.026	8
LOC103314980	uncharacterized	3.099	2.720	1.167	1.442	0.024	2
LOC664463	cytochrome P450 6BQ12	4.914	4.791	3.022	2.944	0.006	4
LOC103315156	toll-like receptor 3	3.437	3.067	1.557	1.675	0.001	2

LOC657454	probable cytochrome P450 9f2	4.346	3.952	2.610	2.383	0.005	3
LOC656825	peroxiredoxin-6	9.720	9.138	8.018	7.827	0.004	2
LOC100142488	uncharacterized	3.460	3.899	1.826	1.600	0.037	6
LOC660938	glucose dehydrogenase [FAD, quinone]	6.708	6.804	5.077	5.089	0.035	6
LOC661455	venom acid phosphatase Acph-1	4.372	3.980	2.744	1.633	0.047	2
LOC660846	UDP-glucuronosyltransferase 2C1-like	3.800	3.214	2.225	1.887	0.002	9
LOC664473	probable cytochrome P450 6BQ6	2.859	3.118	1.354	1.456	0.031	4
LOC103312412	protein toll-like	6.185	5.710	4.712	4.475	0.028	3
LOC658359	multidrug resistance-associated protein 4-like	2.825	2.567	1.373	1.289	0.044	5
LOC656545	amidophosphoribosyltransferase-like	8.923	8.239	7.491	7.822	0.014	10
LOC661478	facilitated trehalose transporter Tret1-like	2.460	2.657	1.056	0.594	0.020	9
LOC657967	uncharacterized	5.387	5.752	4.016	4.129	0.001	5
CYP346B2	cytochrome P450 346B2	4.175	4.049	2.820	2.467	0.011	5
LOC659208	uncharacterized	2.867	3.667	1.575	2.530	0.029	un
LOC658727	L-galactose dehydrogenase	6.040	5.979	4.781	4.937	0.008	5
LOC657479	calcitonin gene-related peptide type 1 receptor	2.575	2.099	1.360	0.661	0.009	un
LOC100142340	alpha-sarcoglycan isoform X2	3.964	3.951	2.764	2.811	0.015	9
LOC103313188	rho-associated protein kinase 1 isoform X2	3.598	3.476	2.430	2.476	0.043	5
LOC103314909	huntingtin-like	3.031	3.177	1.949	2.189	0.044	un
LOC660688	proline-rich extensin-like protein EPR1	3.477	3.262	2.398	2.295	0.009	un
Tyr1	pro-phenol oxidase subunit 1	5.373	5.068	4.305	4.527	0.037	2
LOC662197	alpha-N-acetylgalactosaminidase-like	5.532	5.450	4.465	4.595	0.021	7
LOC103312143	similar to antibacterial peptide PBSIP	8.077	7.955	7.024	7.055	0.008	8
LOC656657	beta-sarcoglycan	3.745	3.854	2.750	3.034	0.007	2
LOC103312986	testicular acid phosphatase homolog	4.094	3.733	3.102	2.907	0.027	5
LOC664309	chaoptic-like protein	2.928	2.790	1.953	2.184	0.004	3
LOC664606	intraflagellar transport protein 52 homolog	4.314	4.497	3.370	3.453	0.011	7
LOC100141658	probable multidrug resistance-associated protein lethal(2)03659	6.548	6.697	5.613	5.575	0.005	6
LOC663932	venom dipeptidyl peptidase 4	6.708	6.928	5.792	5.661	0.001	5
LOC103312835	probable methyltransferase-like protein 15 homolog	5.630	5.658	4.723	4.797	0.005	7
LOC659954	dihydrolipoyllysine-residue succinyltransferase component of 2-oxoglutarate dehydrogenase complex, mitochondrial	8.702	8.831	7.823	7.921	0.001	7
LOC657250	neutral alpha-glucosidase AB-like	3.109	3.116	2.230	2.124	0.020	3
LOC661860	nucleoplasmin-like protein isoform X2	9.080	8.974	8.238	8.347	0.021	9
LOC661507	cytochrome P450 9AA1	4.481	4.447	3.650	3.298	0.042	8
LOC103314059	kielin/chordin-like protein	4.210	4.059	3.417	3.775	0.022	8

LOC661174	rho-related BTB domain-containing protein 2, transcript variant X2	5.352	5.390	4.640	4.964	0.037	9
Cyp4g7	cytochrome P450 monooxygenase CYP4G7	7.803	7.510	7.093	6.904	0.046	4
LOC662650	isoaspartyl peptidase/L-asparaginase	6.799	6.735	6.121	6.157	0.014	8
LOC103314229	feline leukemia virus subgroup C receptor-related protein 2-like	3.270	3.306	2.592	2.402	0.046	9
LOC656501	splicing factor 3B subunit 4	5.844	5.647	5.200	5.162	0.002	3
LOC660182	rhophilin-2 isoform X4	5.483	5.345	4.851	4.839	0.013	7
LOC660285	locomotion-related protein Hikaru genki	3.754	3.833	3.130	3.275	0.035	3
LOC655898	pre-mRNA-splicing ATP-dependent RNA helicase PRP28-like	6.519	6.463	5.898	6.049	0.023	9
LOC657528	dihydrolipoyl dehydrogenase, mitochondrial	7.432	7.481	6.813	6.638	0.004	un
LOC100141906	uncharacterized	9.085	9.018	8.472	8.453	0.045	2
LOC103313823	ataxin-1	6.098	5.978	5.527	5.993	0.005	3
LOC100141859	zinc finger protein OZF	2.894	3.134	2.333	2.439	0.013	2
LOC657073	delta(24)-sterol reductase	5.095	4.785	4.541	4.397	0.038	7
LOC661517	protein HID1	5.141	5.114	4.624	4.383	0.001	8
LOC657538	aminoacyl tRNA synthase complex-interacting multifunctional protein 1	7.808	7.797	7.347	7.019	0.014	un
LOC660199	WW domain-binding protein 4 isoform X2	4.935	5.225	4.502	4.802	0.045	9
Ago-2a	Argonaute-2a	3.709	3.731	3.319	3.320	0.049	X
LOC664394	DNA methyltransferase 1-associated protein 1	4.443	4.570	4.080	4.199	0.041	7
LOC662487	neutral and basic amino acid transport protein rBAT	5.945	5.854	5.588	5.558	0.038	7
LOC658169	tRNA-splicing ligase RtcB homolog	5.422	5.498	5.075	4.930	0.024	9
LOC656634	COP9 signalosome complex subunit 7b isoform X3	5.858	6.213	5.517	5.032	0.021	9
LOC657072	26S proteasome non-ATPase regulatory subunit 14	8.239	8.243	7.916	8.234	0.020	8
LOC663708	actin-binding protein IPP	6.028	6.197	5.719	5.779	0.039	9
LOC659404	uncharacterized	3.711	3.790	3.420	2.959	0.008	8
LOC663003	uncharacterized	5.557	5.716	5.270	5.174	0.049	9
LOC656520	uncharacterized	3.235	3.595	3.005	3.203	0.031	6
LOC100142339	zinc finger protein OZF-like	4.470	4.387	4.353	4.098	0.027	6
LOC663293	heat shock protein 70 A1	1.696	0.874	1.585	2.023	0.016	7
LOC662522	factor VIII intron 22 protein-like	4.114	4.441	4.006	3.678	0.027	8
LOC661398	DNA replication ATP-dependent helicase/nuclease DNA2	2.687	2.671	2.596	2.269	0.035	3
LOC661136	protein ST7 homolog	4.939	5.152	4.885	4.736	0.013	10
LOC103313179	G2/mitotic-specific cyclin-B	5.824	5.578	5.772	5.112	0.037	5
LOC664524	histone-lysine N-methyltransferase setd3	4.787	4.746	4.748	4.295	0.035	4

7
