# Supplementary material for: Genes related to mitochondrial functions are differentially expressed in phosphine-resistant and -susceptible Tribolium castaneum
Source: BMC Genomics. 2015 Nov 18;16:968. doi: 10.1186/s12864-015-2121-0 (PMC4650509; doi:10.1186/s12864-015-2121-0)
Supplement: Additional file 5: — Relative coverage of transcripts from susceptible (A, B) and phosphine-resistant (C, D) T. castaneum adults from this study, either not exposed (A, C) or exposed (B, D) to phosphine, aligning to DLD mRNA from a phosphine-susceptible T. castaneum adult strain (QTC4, accession JX434604) using SeqMan Pro (DNAStar). Thin red line – single strand coverage; thick red line – exceeds threshold of coverage; thin green – coverage on both strands; thick green – above threshold coverage; thin cyan – single direction coverage only. (PDF 99 kb) [file 12864_2015_2121_MOESM6_ESM.pdf]

Additional File 4. Relative coverage of transcripts from susceptible (A, B) and phosphine-resistant (C, D) *T. castaneum* adults from this study, either not exposed (A, C) or exposed (B, D) to phosphine, aligning to DLD mRNA from a phosphine-susceptible *T. castaneum* adult strain (QTC4, accession JX434604) using SeqMan Pro (DNAStar). Thin red line – single strand coverage; thick red line – exceeds threshold of coverage; thin green – coverage on both strands; thick green – above threshold coverage; thin cyan – single direction coverage only.

A.

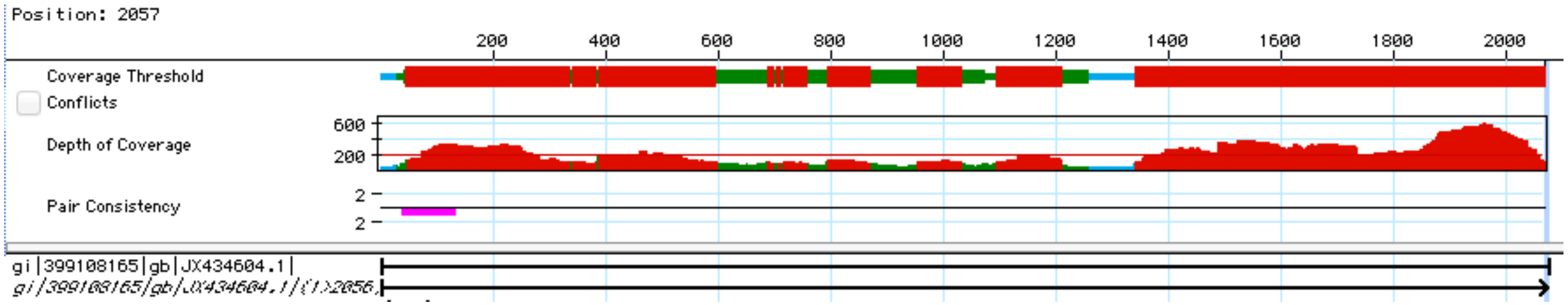

B.

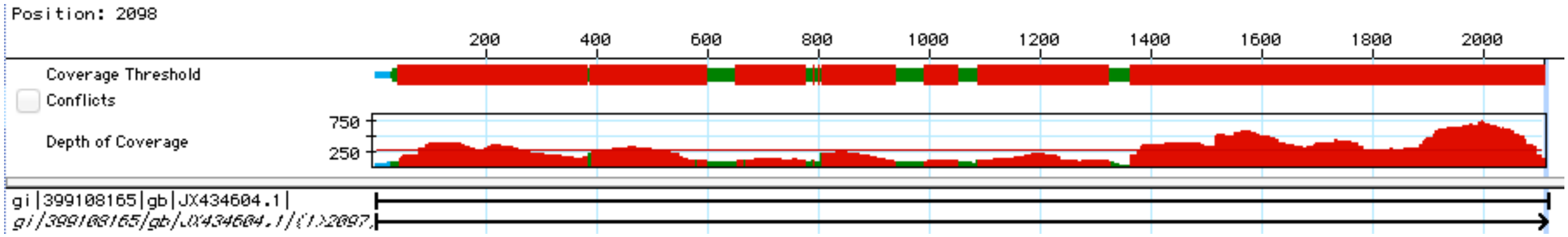

C.

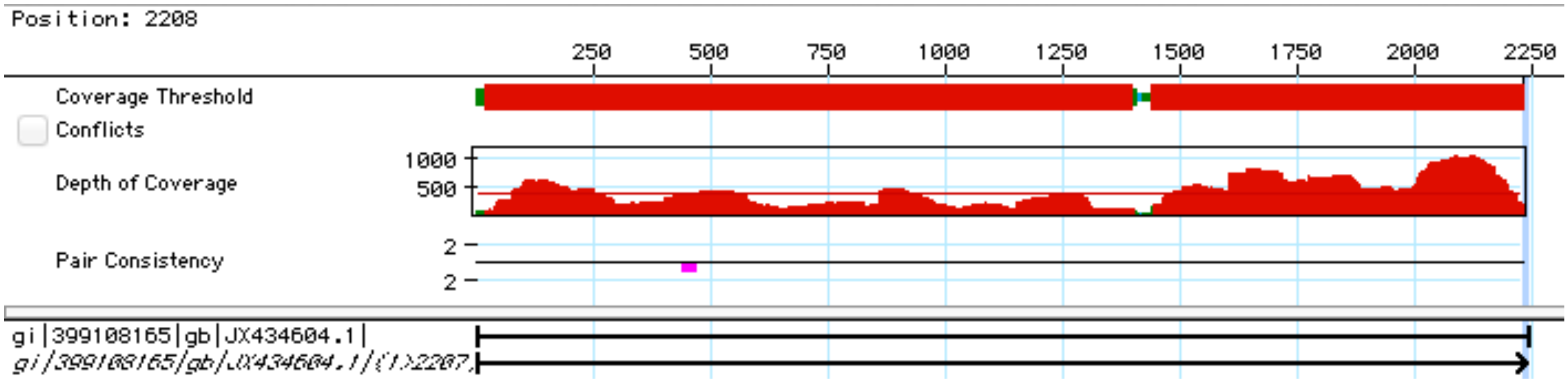
Position: 2208
250
500
750
1000
1250
1500
1750
2000
2250
Coverage Threshold
Conflicts
1000
500
Depth of Coverage
2
Pair Consistency
2
gi|399108165|gb|JX434604.1|
gi|399108165|gb|JX434604.1|(1>2207)

D.

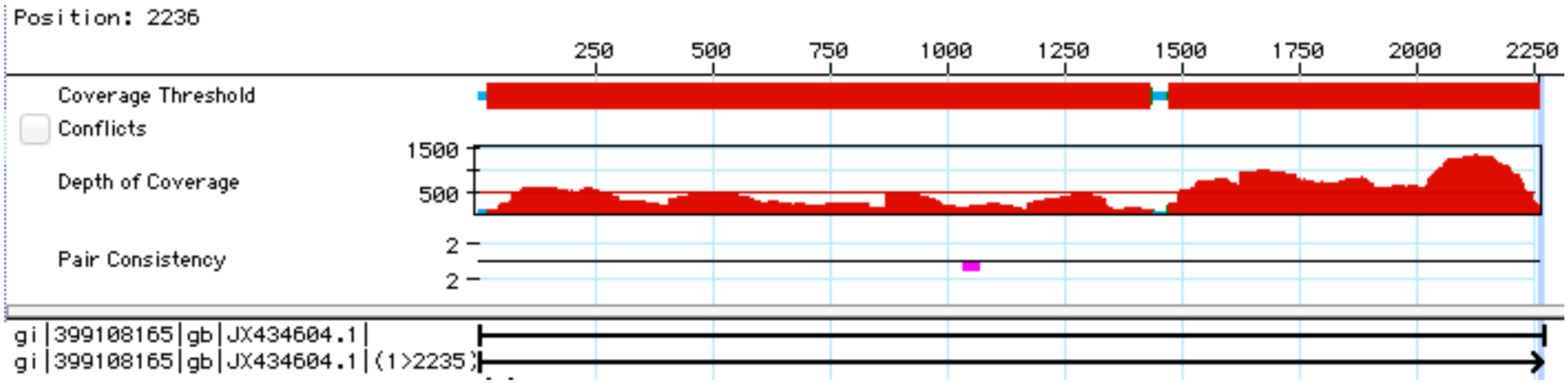
Position: 2236
250
500
750
1000
1250
1500
1750
2000
2250
Coverage Threshold
Conflicts
1500
500
Depth of Coverage
2
Pair Consistency
2
gi|399108165|gb|JX434604.1|
gi|399108165|gb|JX434604.1|(1>2235)
