# Supplementary material for: Genes related to mitochondrial functions are differentially expressed in phosphine-resistant and -susceptible Tribolium castaneum
Source: BMC Genomics. 2015 Nov 18;16:968. doi: 10.1186/s12864-015-2121-0 (PMC4650509; doi:10.1186/s12864-015-2121-0)
Supplement: Additional file 6: — Multiple sequence alignment of RNA-Seq transcriptome data from phosphine-susceptible (TcPhosSusUxp_DLD, TcPhosSusExp_DLD) and -resistant (TcPhosResUxp_DLD, TcPhosResExp_DLD) T. castaneum to DLD mRNA from other strains. QTC sequences corresponded to gi|399108165|gb|JX434604.1| (QTC4); gi|399108167|gb|JX434605.1| (QTC931); gi|399108169|gb|JX434606.1| (QTC1012); and gi|399108171|gb|JX434607.1| (QTC1389) [10]. R = purine (A or G); M = Amino (A or C); S = Strong (G or C); Y = pyrimidine (C or T); - = deletion; lower case letters indicate polymorphism (most common nucleotide shown). Shaded are recognition sequences for restriction enzymes that may be used to detect resistance, and sites are identified by arrows. (PDF 100 kb) [file 12864_2015_2121_MOESM7_ESM.pdf]

Additional File 5. Multiple sequence alignment of RNA-Seq transcriptome data from phosphine-susceptible (TcPhosSusUxp_DLD, TcPhosSusExp_DLD) and -resistant (TcPhosResUxp_DLD, TcPhosResExp_DLD) *T. castaneum* to DLD mRNA from other strains. QTC sequences corresponded to gi|399108165|gb|JX434604.1| (QTC4); gi|399108167|gb|JX434605.1| (QTC931); gi|399108169|gb|JX434606.1| (QTC1012); and gi|399108171|gb|JX434607.1| (QTC1389) [10]. R = purine (A or G); M = Amino (A or C); S = Strong (G or C); Y = pyrimidine (C or T); - = deletion; lower case letters indicate polymorphism (most common nucleotide shown). Shaded are recognition sequences for restriction enzymes that may be used to detect resistance, and sites are identified by arrows.

```
QTC4                GTCAGCTGTCATTATTATTTAAGAAAATTTATTTAAAAAGATGCAATCGGCCATTCGAAA
TcPhosSusExp_DLD    GTCAGCTGTCATTATTATTTAAGAAAATTTATTTAAAAAGATGCAATCGGCCATTCGAAA
QTC931              ---------------------AGAAAATTTATTTAAAAAGATGCAATCGGCCATTCGAAA
QTC1012             ------------------------------------------------------------
QTC1389             ------------------------------------------------------------
TcPhosResExp_DLD    GTCAGCTGTCATTATTATTTAAGAAAATTTATTTAAAAAGATGCAATCGGCCATTCGAAA
TcPhosSusUxp_DLD    GTCAGCTGTCATTATTATTTAAGAAAATTTATTTAAAAAGATGCAATCGGCCATTCGAAA
TcPhosResUxp_DLD    GTCAGCTGTCATTATTATTTAAGAAAATTTATTTAAAAAGATGCAATCGGCCATTCGAAA

QTC4                CGTTGTCTCCTCGTCACTTAAGATACGGTGCAATCGGGGGGCCCTGACTGTCTTCCACCA
TcPhosSusExp_DLD    CGTTGTCTCCTCGTCACTTAAGATACGGTGCAATCGGGGGGCCCTGACTGTCTTCCACCA
QTC931              CGTTGTCTCCTCGTCACTTAAGATACGGTGCAATCGGGGGGCCCTGACTGTCTTCCACCA
QTC1012             -----------------TTAAGATACGGTGCAATCGGGGGGCCCTGACTGTCTTCCACCA
QTC1389             --------------------AGATACGGTGCAATCGGGGGGCCCTGACTGTCTTCCACCA
TcPhosResExp_DLD    CGTTGTCTCCTCGTCACTTAAGATACGGTGCAATCGGGGGGCCCTGACTGTCTTCCACCA
TcPhosSusUxp_DLD    CGTTGTCTCCTCGTCACTTAAGATACGGTGCAATCGGGGGGCCCTGACTGTCTTCCACCA
TcPhosResUxp_DLD    CGTTGTCTCCTCGTCACTTAAGATACGGTGCAATCGGGGGGCCCTGACTGTCTTCCACCA
                                        ****************************************
                                                                          ↓ SetI
QTC4                CCGTCAATACTCCACAACTCACGATGCGGATTTGGTCGTGATTGGGTCGGGACCTGGGGG
TcPhosSusExp_DLD    CCGTCAATACTCCACAACTCACGATGCGGATTTGGTCGTGATTGGGTCGGGACCTGGGGG
QTC931              CCGTCAATACTCCACAACTCACGATGCGGATTTGGTCGTGATTGGGTCGGGACCTGGGGG
QTC1012             CCGTCAATACTCCACAACTCACGATGCGGATTTGGTCGTGATTGGGTCGGGACCTGGGGG
QTC1389             CCGTCAATACTCCACAACTCACGATGCGGATTTGGTCGTGATTGGGTCGGGACCTGGGGG
TcPhosResExp_DLD    CCGTCAATACTCCACAACTCACGATGCGGATTTGGTCGTGATTGGGTCGGGAYCTGGGGg
TcPhosSusUxp_DLD    CGGTCAATACTCCACAACTCACGATGCGGATTTGGTCGTGATTGGGTCGGGACCTGGGGG
TcPhosResUxp_DLD    CCGTCAATACTCCACAACTCACGATGCGGATTTGGTCGTGATTGGGTCGGGATCTGGGGg
                    * ************************************************** ******
```

```
QTC4                CTACGTTGCGTCGATTAAAGCCGCCCAACTTGGCTTAAAAACAGTATGTATAGAGAAAGA
TcPhosSusExp_DLD    CTACGTTGCGTCGATTAAAGCCGCCCAACTTGGCTTAAAAACAGTATGTATAGAGAAAGA
QTC931              CTACGTTGCGTCGATTAAAGCCGCCCAACTTGGCTTAAAAACAGTATGTATAGAGAAAGA
QTC1012             CTACGTTGCGTCGATTAAAGCCGCCCAACTTGGCTTAAAAACAGTATGTATAGAGAAAGA
QTC1389             CTACGTTGCGTCGATTAAAGCCGCCCAACTTGGCTTAAAAACAGTATGTATAGAGAAAGA
TcPhosResExp_DLD    CTACGTTGCGTCGATTAAAGCYGCCCAACTTGGMTTAAAAACAGTATGTATAGAGAAAGA
TcPhosSusUxp_DLD    CTACGTTGCGTCGATTAAAGCYGCCCAACTTGGCTTAAAAACAGTATGTATAGAGAAAGA
TcPhosResUxp_DLD    CTACGTTGCGTCGATTAAAGCCGCCCAACTTGGCTTAAAAACAGTATGTATAGAGAAAGA
                    ********************* *********** **************************

QTC4                ACCGACTTTGGGGGGCACCTGCCTCAACGTGGGGTGTATACCATCCAAAGCCTTACTCAA
TcPhosSusExp_DLD    ACCGACTTTGGGGGGCACCTGCCTCAACGTGGGGTGTATACCATCCAAAGCCTTACTCAA
QTC931              ACCGACTTTGGGGGGCACTTGTCTCAACGTGGGGTGTATACCATCCAAAGCCTTACTCAA
QTC1012             ACCGACTTTGGGGGGCACTTGTCTCAACGTGGGGTGTATACCATCCAAAGCCTTACTCAA
QTC1389             ACCGACTTTGGGGGGCACTTGTCTCAACGTGGGGTGTATACCATCCAAAGCCTTACTCAA
TcPhosResExp_DLD    ACCGACTTTGGGGGGCACCTGCCTCAACGTGGGGTGTATACCATCCAAAGCCTTACTCAA
TcPhosSusUxp_DLD    ACCGACTTTGGGGGGCACCTGCCTCAACGTGGGGTGTATACCATCCAAAGCCTTACTCAA
TcPhosResUxp_DLD    ACCGACTTTGGGGGGCACYTGCCTCAACGTGGGGTGTATACCATCCAAAGCCTTACTCAA
                    ****************** ** **************************************

QTC4                CAACTCACACTATTACCACATGGCCCATTCCGGTGACCTGGGGGCACGTGGCATAAGCGT
TcPhosSusExp_DLD    CAACTCACACTATTACCACATGGCCCATTCCGGTGACCTGGGGGCACGTGGCATAAGCGT
QTC931              CAACTCACACTATTACCACATGGCCCATTCCGGTGACCTGGGGGCACGTGGCATAAGCGT
QTC1012             CAACTCACACTATTACCACATGGCCCATTCCGGTGACCTGGGGGCACGTGGCATAAGCGT
QTC1389             CAACTCACACTATTACCACATGGCCCATTCCGGTGACCTGGGGGCACGTGGCATAAGCGT
TcPhosResExp_DLD    CAACTCACACTATTACCACATGGCCCATTCCGGTGACCTGGGGGCACGTGGCATAAGCGT
TcPhosSusUxp_DLD    CAACTCACACTATTACCACATGGCCCATTCCGGTGACCTGGGGGCACGTGGCATAAGCGT
TcPhosResUxp_DLD    CAACTCACACTATTACCACATGGCCCATTCCGGTGACCTGGGGGCACGTGGCATAAGCGT
                    ************************************************************
                                      Rla1
QTC4                GGACAATGTCCGACTTGACTTGGATAAATTAATGGGGCAAAGGAAAATGCTGTCAAGGC
TcPhosSusExp_DLD    GGACAATGTCCGACTTGACTTGGATAAATTAATGGGGCAAAGGAAAATGCTGTCAAGGC
QTC931              GGACAATGTCCGACTTGATTTGGATAAATTAATGGGGCAAAGGAAAATGCTGTCAAGGC
QTC1012             GGACAATGTCCGACTTGATTTGGATAAATTAATGGGGCAAAGGAAAATGCTGTCAAGGC
QTC1389             GGACAATGTCCGACTTGATTTGGATAAATTAATGGGGCAAAGGAAAATGCTGTCAAGGC
TcPhosResExp_DLD    GGACAATGTCCGACTTGAYTTGGATAAATTAATGGGGCAAAGGAAAATGCTGTCAAGGC
TcPhosSusUxp_DLD    GGACAATGTCCGACTTGATTTGGATAAATTAATGGGGCAAAGGAAAATGCTGTCAAGGC
TcPhosResUxp_DLD    GGACAATGTCCGACTTGAYTTGGATAAATTAATGGGGCAAAGGAAAATGCTGTCAAGGC
                    ****************** *****************************************

QTC4                TTTAACTGGGGGCATAGCGCAACTTTTCAAAAAGAATAAAGTCACACTGATCAACGGACA
TcPhosSusExp_DLD    TTTAACTGGGGGCATAGCGCAACTTTTCAAAAAGAATAAAGTCACACTGATCAACGGACA
QTC931              TTTAACTGGAAGCATAGCGCAACTTTTCAAAAAGAATAAAGTCACACTGATCAACGGACA
QTC1012             TTTAACTGGGGGCATAGCGCAACTTTTCAAAAAGAATAAAGTCACACTGATCAACGGACA
QTC1389             TTTAACTGGAGGCATAGCGCAACTTTTCAAAAAGAATAAAGTCACACTGATCAACGGACA
TcPhosResExp_DLD    TTTAACTGGRGGCATAGCGCAACTTTTCAAAAAGAATAAAGTCACACTSATCAACGGACA
TcPhosSusUxp_DLD    TTTAACTGGRGGCATAGCGCAACTTTTCAAAAAGAATAAAGTCACACTSATCAACGGACA
TcPhosResUxp_DLD    TTTAACTGGRGGCATAGCGCAACTTTTCAAAAAGAATAAAGTCACACTSATCAACGGACA
                    ********* .*************************************.***********

QTC4                TGGGAAAATTACAGGTGTTAATCAAGTGACTGCTTTAAAACCGGATGGATCGTCGGAAGT
TcPhosSusExp_DLD    TGGGAAAATTACAGGTGTTAATCAAGTGACTGCTTTAAAACCGGATGGATCGTCGGAAGT
QTC931              TGGGAAAATTACAGGTGTTAATCAAGTGACTGCTTTAAAACCGGATGGATCGTCGGAAGT
QTC1012             TGGGAAAATTACAGGTGTTAATCAAGTGACTGCTTTAAAACCGGATGGATCGTCGGAAGT
QTC1389             TGGGAAAATTACAGGTGTTAATCAAGTGACTGCTTTAAAACCGGATGGATCGTCGGAAGT
TcPhosResExp_DLD    TGGGAAAATTACAGGTGTTAATCAAGTGACTGCTTTAAAACCGGATGGATCGTCGGAAGT
TcPhosSusUxp_DLD    TGGGAAAATTACAGGTGTTAATCAAGTGACTGCTTTAAAACCGGATGGATCGTCGGAAGT
TcPhosResUxp_DLD    TGGGAAAATTACAGGTGTTAATCAAGTGACTGCTTTAAAACCGGATGGATCGTCGGAAGT
                    ************************************************************

QTC4                TGTTAATACGAAAAATGTGCTAATTGCCACTGGGTCAGAAGTTACTCCATTTCCTGGAAT
TcPhosSusExp_DLD    TGTTAATACGAAAAATGTGCTAATTGCCACTGGGTCAGAAGTTACTCCATTTCCTGGAAT
QTC931              TGTTAATACGAAAAATGTGCTAATTGCCACTGGGTCAGAAGTTACTCCATTTCCTGGAAT
QTC1012             TGTTAATACGAAAAATGTGCTAATTGCCACTGGGTCAGAAGTTACTCCATTTCCTGGAAT
QTC1389             TGTTAATACGAAAAATGTGCTAATTGCCACTGGGTCAGAAGTTACTCCATTTCCTGGAAT
TcPhosResExp_DLD    TGTTAATACGAAAAATGTGCTAATTGCCACTGGGTCAGAAGTTACTCCATTTCCTGGAAT
TcPhosSusUxp_DLD    TGTTAATACGAAAAATGTGCTAATTGCCACTGGGTCAGAAGTTACTCCATTTCCTGGAAT
TcPhosResUxp_DLD    TGTTAATACGAAAAATGTGCTAATTGCCACTGGGTCAGAAGTTACTCCATTTCCTGGAAT
                    ************************************************************
```

```
QTC4                  TGAAATCGACGAGGAACAAATCGTTTCATCAACTGGCGCTCTTTCATTGAAAGAAGTCCC
TcPhosSusExp_DLD      TGAAATCGACGAGGAACAAATCGTTTCATCAACTGGCGCTCTTTCATTGAAAGAAGTCCC
QTC931                TGAAATCGACGAGGAACAAATCGTTTCATCAACTGGCGCTCTTTCATTGAAAGAAGTCCC
QTC1012               TGAAATCGACGAGGAACAAATCGTTTCATCAACTGGCGCTCTTTCATTGAAAGAAGTCCC
QTC1389               TGAAATCGACGAGGAACAAATCGTTTCATCAACTGGCGCTCTTTCATTGAAAGAAGTCCC
TcPhosResExp_DLD      TGAAATCGACGAGGAACAAATCGTTTCATCAACTGGCGCTCTTTCATTGAAAGAAGTCCC
TcPhosSusUxp_DLD      TGAAATCGACGAGGAACAAATCGTTTCATCAACTGGCGCTCTTTCATTGAAAGAAGTCCC
TcPhosResUxp_DLD      TGAAATCGACGAGGAACAAATCGTTTCATCAACTGGCGCTCTTTCATTGAAAGAAGTCCC
                      ************************************************************

QTC4                  TAAACGACTAATTGTAATCGGCGCCGGTGTAATCGGCCTTGAATTAGGCTCAGTTTGGTC
TcPhosSusExp_DLD      TAAACGACTAATTGTAATCGGCGCCGGTGTAATCGGCCTTGAATTAGGCTCAGTTTGGTC
QTC931                TAAACGACTAATTGTAATCGGCGCCGGTGTAATCGGCCTTGAATTAGGCTCAGTTTGGTC
QTC1012               TAAACGACTAATTGTAATCGGCGCCGGTGTAATCGGCCTTGAATTAGGCTCAGTTTGGTC
QTC1389               TAAACGACTAATTGTAATCGGCGCCGGTGTAATCGGCCTTGAATTAGGCTCAGTTTGGTC
TcPhosResExp_DLD      TAAACGACTAATTGTAATCG-CGCCGGTGTAATCGGCCTTGAATTAGGCTCAGTTTGGTC
TcPhosSusUxp_DLD      TAAACGACTAATTGTAATCG-CGCCGGTGTAATCGGCCTTGAATTAGGCTCAGTTTGGTC
TcPhosResUxp_DLD      TAAACGACTAATTGTAATCG-CGCCGGTGTAATCGGCCTTGAATTAGGCTCAGTTTGGTC
                      ******************** ***************************************

QTC4                  TCGATTGGGTTCCGAAGTGACCGCTGTAGAATTCTTAAGCAGTATCGGAGGTGTAGGCAT
TcPhosSusExp_DLD      TCGATTGGGTTCCGAAGTGACCGCTGTAGAATTCTTAAGCAGTATCGGAGGTGTAGGCAT
QTC931                TCGATTGGGTTCCGAAGTGACCGCTGTAGAATTCTTAAGCAGTATCGGAGGTGTAGGCAT
QTC1012               TCGATTGGGTTCCGAAGTGACCGCTGTAGAATTCTTAAGCAGTATCGGAGGTGTAGGCAT
QTC1389               TCGATTGGGTTCCGAAGTGACCGCTGTAGAATTCTTAAGCAGTATCGGAGGTGTAGGCAT
TcPhosResExp_DLD      TCGATTGGGTTCCGAAGTGACCGCTGTAGAATTCTTAAGCAGTATCGGAGGTGTAGGCAT
TcPhosSusUxp_DLD      TCGATTGGGTTCCGAAGTGACCGCTGTAGAATTCTTAAGCAGTATCGGAGGTGTAGGCAT
TcPhosResUxp_DLD      TCGATTGGGTTCCGAAGTGACCGCTGTAGAATTCTTAAGCAGTATCGGAGGTGTAGGCAT
                      ************************************************************

QTC4                  CGATGGCGAAGTGGCTAAAACACTACAAAAAGTATTAACCAAACAAGGATTAAAATTCAA
TcPhosSusExp_DLD      CGATGGCGAAGTGGCTAAAACACTACAAAAAGTATTAACCAAACAAGGATTAAAATTCAA
QTC931                TGATGGCGAAGTGGCTAAAACACTACAAAAAGTATTAACCAAACAAGGATTAAAATTCAA
QTC1012               CGATGGCGAAGTGGCTAAAACACTACAAAAAGTATTAACCAAACAAGGATTAAAATTCAA
QTC1389               TGATGGCGAAGTGGCTAAAACACTACAAAAAGTATTAACCAAACAAGGATTAAAATTCAA
TcPhosResExp_DLD      CGATGGCGAAGTGGCTAAAACACTACAAAAAGTATTAACCAAACAAGGATTAAAATTCAA
TcPhosSusUxp_DLD      CGATGGCGAAGTGGCTAAAACACTACAAAAAGTATTAACCAAACAAGGATTAAAATTCAA
TcPhosResUxp_DLD      CGATGGCGAAGTGGCTAAAACACTACAAAAAGTATTAACCAAACAAGGATTAAAATTCAA
                       ***********************************************************

QTC4                  ATTGGGCACAAAAGTAACAGCCGCCCAAAAATCGGGAGGAGTTGTTAAAGTCAGCATTGA
TcPhosSusExp_DLD      ATTGGGCACAAAAGTAACAGCCGCCCAAAAATCGGGAGGAGTTGTTAAAGTCAGCATTGA
QTC931                ATTGGGCACGAAAGTAACAGCCGCCCAAAAATCGGGAGGAGTTGTTAAAGTCAGCATTGA
QTC1012               ATTGGGCACGAAAGTAACAGCCGCCCAAAAATCGGGAGGAGTTGTTAAAGTCAGCATTGA
QTC1389               ATTGGGCACGAAAGTAACAGCCGCCCAAAAATCGGGAGGAGTTGTTAAAGTCAGCATTGA
TcPhosResExp_DLD      ATTGGGCACGAAAGTAACAGCCGCCCAAAAATCGGGAGGAGTTGTTAAAGTCAGCATTGA
TcPhosSusUxp_DLD      ATTGGGCACRAAAGTAACAGCCGCCCAAAAATCGGGAGGAGTTGTTAAAGTCAGCATTGA
TcPhosResUxp_DLD      ATTGGGCACGAAAGTAACAGCCGCCCAAAAATCGGGAGGAGTTGTTAAAGTCAGCATTGA
                      ********* **************************************************

QTC4                  AGATGCGAAAAATCCCGACAAGAAAGAAGAGTTGGAATGTGAAGTATTGTTAGTTTGTGT
TcPhosSusExp_DLD      AGATGCGAAAAATCCCGACAAGAAAGAAGAGTTGGAATGTGAAGTATTGTTAGTTTGTGT
QTC931                AGATGCGAAAAATCCCGACAAGAAAGAAGAGTTGGAATGTGAAGTGTTGTTAGTTTGTGT
QTC1012               AGATGCGAAAAATCCCGACAAGAAAGAAGAGTTGGAATGTGAAGTATTGTTAGTTTGTGT
QTC1389               AGATGCGAAAAATCCCGACAAGAAAGAAGAGTTGGAATGTGAAGTATTGTTAGTTTGTGT
TcPhosResExp_DLD      AGATGCGAAAAATCCCGACAAGAAAGAAGAGTTGGAATGTGAAGTATTGTTAGTTTGTGT
TcPhosSusUxp_DLD      AGATGCGAAAAATCCCGACAAGAAAGAAGAGTTGGAATGTGAAGTATTGTTAGTTTGTGT
TcPhosResUxp_DLD      AGATGCGAAAAATCCCGACAAGAAAGAAGAGTTGGAATGTGAAGTATTGTTAGTTTGTGT
                      *********************************************.**************

QTC4                  TGGACGCAGACCTTACACTCATAATCTTGGTCTTGAAGAGATGGGGATTGAAAGGGACCA
TcPhosSusExp_DLD      TGGACGCAGACCTTACACTCATAATCTTGGTCTTGAAGAGATGGGGATTGAAAGGGACCA
QTC931                TGGACGCAGACCTTACACTCATAATCTTGGTCTTGAAGAGATGGGGATTGAAAGGGACCA
QTC1012               TGGACGCAGACCTTACACTCATAATCTTGGTCTTGAAGAGATGGGGATTGAAAGGGACCA
QTC1389               TGGACGCAGACCTTACACTCATAATCTTGGTCTTGAAGAGATGGGGATTGAAAGGGACCA
TcPhosResExp_DLD      TGGACGCAGACCTTACACTCATAATCTTGGTCTTGAAGAGATGGGAATTGAAAGGGACCA
TcPhosSusUxp_DLD      TGGACGCAGACCTTACACTCATAATCTTGGTCTTGAAGAGATGGGAATTGAAAGGGACCA
TcPhosResUxp_DLD      TGGACGCAGACCTTACACTCATAATCTTGGTCTTGAAGAGATGGGAATTGAAAGGGACCA
                      *********************************************.**************
```

```
                        ↓ HpyAV
QTC4                    GAAAGGAAGGATTCCAGTCAATTCACATTTCCAGACTGTTATTCCTAATATTCATGCGAT
TcPhosSusExp_DLD        GAAAGGAAGGATTCCAGTCAATTCACATTTCCAGACTGTTATTCCTAATATTCATGCGAT
QTC931                  GAAAGGGAGGATTCCAGTCAATTCACATTTCCAGACTGTTATTCCTAATATTCATGCGAT
QTC1012                 GAAAGGGAGGATTCCAGTCAATTCACATTTCCAGACTGTTATTCCTAATATTCATGCGAT
QTC1389                 GAAAGGGAGGATTCCAGTCAATTCACATTTCCAGACTGTTATTCCTAATATTCATGCGAT
TcPhosResExp_DLD        GAAAGGGAGGATTCCAGTCAATTCACATTTCCAGACTGTTATTCCTAATATTCATGCGAT
TcPhosSusUxp_DLD        GAAAGGAAGGATTCCAGTCAATTCACATTTCCAGACTGTTATTCCTAATATTCATGCGAT
TcPhosResUxp_DLD        GAAAGGGAGGATTCCAGTCAATTCACATTTCCAGACTGTTATTCCTAATATTCATGCGAT
                        ******.*****************************************************

QTC4                    AGGGGATTGTATTCATGGACCTATGTTGGCACACAAAGCCGAAGATGAAGGCATCATTTG
TcPhosSusExp_DLD        AGGGGATTGTATTCATGGACCTATGTTGGCACACAAAGCCGAAGATGAAGGCATCATTTG
QTC931                  AGGGGATTGTATTCATGGACCTATGTTGGCACACAAAGCCGAAGATGAAGGCATCATTTG
QTC1012                 AGGGGATTGTATTCATGGACCTATGTTGGCACACAAAGCCGAAGATGAAGGCATCATTTG
QTC1389                 AGGGGATTGTATTCATGGACCTATGTTGGCACACAAAGCCGAAGATGAAGGCATCATTTG
TcPhosResExp_DLD        AGGGGATTGTATTCATGGACCTATGTT-GCACACAAAGCCGAAGATGAAGgCATCATTTG
TcPhosSusUxp_DLD        AGGGGATTGTATTCATGGACCTATGTT-GCACACAAAGCCGAAGATGAAGgCATCATTTG
TcPhosResUxp_DLD        AGGGGATTGTATTCATGGACCTATGTT-GCACACAAAGCCGAAGATGAAGgCATCATTTG
                        *************************** ********************** *********

QTC4                    CGTGGAGGGAATCACCGGTGGTCCCGTCCACATCGACTACAACTGTGTCCCATCTGTAAT
TcPhosSusExp_DLD        CGTGGAGGGAATCACCGGTGGTCCCGTCCACATCGACTACAACTGTGTCCCATCTGTAAT
QTC931                  CGTGGAGGGAATCACCGGTGGTCCCGTCCACATCGACTACAACTGTGTCCCATCTGTAAT
QTC1012                 CGTGGAGGGAATCACCGGTGGTCCCGTCCACATCGACTACAACTGTGTCCCATCTGTAAT
QTC1389                 CGTGGAGGGAATCACCGGTGGTCCCGTCCACATCGACTACAACTGTGTCCCATCTGTAAT
TcPhosResExp_DLD        CGTGGAGGGAATCACCGGTGGTCCCGTCCACATCGACTACAACTGTGTCCCATCTGTAAT
TcPhosSusUxp_DLD        CGTGGAGGGAATCACCGGTGGTCCCGTCCACATCGACTACAACTGTGTCCCATCTGTAAT
TcPhosResUxp_DLD        CGTGGAGGGAATCACCGGTGGTCCCGTCCACATCGACTACAACTGTGTCCCATCTGTAAT
                        ************************************************************

QTC4                    CTACACTCATCCTGAAGTAGGCTGGGTTGGACGTAGTGAAGAAGACTTAAAAAGCGAAGG
TcPhosSusExp_DLD        CTACACTCATCCTGAAGTAGGCTGGGTTGGACGTAGTGAAGAAGACTTAAAAAGCGAAGG
QTC931                  CTACACTCATCCTGAAGTGGGCTGGGTTGGACGTAGTGAAGAGGACTTAAAAAGCGAAGG
QTC1012                 CTACACTCATCCTGAAGTAGGCTGGGTTGGACGTAGTGAAGAGGACTTAAAAAGCGAAGG
QTC1389                 CTACACTCATCCTGAAGTAGGCTGGGTTGGACGTAGTGAAGAAGACTTAAAAAGCGAAGG
TcPhosResExp_DLD        CTACACTCATCCTGAAGTAGGCTGGGTTGGACGTAGTGAAGAAGACTTAAAAAGCGAAGG
TcPhosSusUxp_DLD        CTACACTCATCCTGAAGTAGGCTGGGTTGGACGTAGTGAAGAAGACTTAAAAAGCGAAGG
TcPhosResUxp_DLD        CTACACTCATCCTGAAGTAGGCTGGGTTGGACGTAGTGAAGAAGACTTAAAAAGCGAAGG
                        ******************.***********************.*****************

QTC4                    AATCGACTACAAAATCGGCAAATTCCCATTCATGGCCAACTCCCGAGCAAAAACCAACAA
TcPhosSusExp_DLD        AATCGACTACAAAATCGGCAAATTCCCATTCATGGCCAACTCCCGAGCAAAAACCAACAA
QTC931                  AATCGACTACAAAACCGGCAAATTCCCATTCATGGCCAACTCCCGAGCAAAAACCAACAA
QTC1012                 AATCGACTACAAAATCGGCAAATTCCCATTCATGGCCAACTCCCGAGCAAAAACCAACAA
QTC1389                 AATCGACTACAAAATCGGCAAATTCCCATTCATGGCCAACTCCCGAGCAAAAACCAACAA
TcPhosResExp_DLD        AATCGACTACAAAATCGGCAAATTCCCATTCATGGCCAACTCCCGAGCAAAAACCAACAA
TcPhosSusUxp_DLD        AATCGACTACAAAATCGGCAAATTCCCATTCATGGCCAACTCCCGAGCAAAAACCAAYAA
TcPhosResUxp_DLD        AATCGACTACAAAATCGGCAAATTCCCATTCATGGCCAACTCCCGAGCAAAAACCAACAA
                        ************** ****************************************** **

QTC4                    CGAAACCGACGGTTTCGTCAAAGTTCTCGCAGATAAAGCAACCGATCGCATTTTAGGCAC
TcPhosSusExp_DLD        CGAAACCGACGGTTTCGTCAAAGTTCTCGCAGATAAAGCAACCGATCGCATTTTAGGCAC
QTC931                  CGAAACCGACGGTTTCGTCAAAGTTCTCGCAGATAAAGCAACCGATCGCATTTTAGGCAC
QTC1012                 CGAAACCGACGGTTTCGTCAAAGTTCTCGCAGATAAAGCAACCGATCGCATTTTAGGCAC
QTC1389                 CGAAACCGACGGTTTCGTCAAAGTTCTCGCAGATAAAGCAACCGATCGCATTTTAGGCAC
TcPhosResExp_DLD        CGAAACCGACGGTTTCGTCAAAGTTCTCGCAGATAAAGCAACCGATCGCATTTTAGGCAC
TcPhosSusUxp_DLD        CGAAACCGACGGTTTCGTCAAAGTTCTCGCAGATAAAGCAACCGATCGCATTTTAGGCAC
TcPhosResUxp_DLD        CGAAACCGACGGTTTCGTCAAAGTTCTCGCAGATAAAGCAACCGATCGCATTTTAGGCAC
                        ************************************************************

QTC4                    TCACATAATCGGGCCCTCTGCCGGCGAATTGATCAACGAAGCGGTGTTAGCACAAGAGTA
TcPhosSusExp_DLD        TCACATAATCGGGCCCTCTGCCGGCGAATTGATCAACGAAGCGGTGTTAGCACAAGAGTA
QTC931                  TCATATAATCGGGCCCTCTGCCGGCGAATTGATCAACGAAGCGGTGTTAGCACAAGAATA
QTC1012                 TCACATAATCGGGCCCTCTGCCGGCGAATTGATCAACGAAGCGGTGTTAGCACAAGAGTA
QTC1389                 TCATATAATCGGGCCCTCTGCCGGCGAATTGATCAACGAAGCGGTGTTAGCACAAGAGTA
TcPhosResExp_DLD        TCAYATAATCGGGCCCTCTGCCGGCGAATTGATCAACGAAGCGGTGTTAGCACAAGAGTA
TcPhosSusUxp_DLD        TCATATAATCGGGCCCTCTGCCGGCGAATTGATCAACGAAGCGGTGTTAGCACAAGAGTA
TcPhosResUxp_DLD        TCAYATAATCGGGCCCTCTGCCGGCGAATTGATCAACGAAGCGGTGTTAGCACAAGAGTA
                        *** *****************************************************.**
```

```
QTC4                 CGGTGCGTCGAGTGAAGATGTCGCTAGAGTGTGTCATGCACATCCCACGTGTGCGGAGGC
TcPhosSusExp_DLD     CGGTGCGTCGAGTGAAGATGTCGCTAGAGTGTGTCATGCACATCCCACGTGTGCGGAGGC
QTC931               CGGAGCGTCGAGTGAAGATGTCGCTAGAGTGTGTCATGCACATCCCACGTGTGCGGAGGC
QTC1012              CGGAGCGTCGAGTGAAGATGTCGCTAGAGTGTGTCATGCACATCCCACGTGTGCGGAGGC
QTC1389              CGGAGCGTCGAGTGAAGATGTCGCTAGAGTGTGTCATGCACATCCCACGTGTGCGGA---
TcPhosResExp_DLD     CGGTGCGTCGAGTGAAGATGTCGCTAGAGTGTGTCATGCACATCCcACGTGTGCGGAG-C
TcPhosSusUxp_DLD     CGGTGCGTCGAGTGAAGATGTCGCTAGAGTGTGTCATGCACATCCcACGTGTGCGGAGgC
TcPhosResUxp_DLD     CGGTGCGTCGAGTGAAGATGTCGCTAGAGTGTGTCATGCACATCCcACGTGTGCGGAGgC
                     ***:***************************************** ***********

QTC4                 CTTAAGAGAG-CCAATTTGGCGTCGTACTTCG-AAAACCGATCA----------------
TcPhosSusExp_DLD     CTTAAGAGAG-CCAATTTGGCGTCGTACTTCG-AAAACCGATCA----------------
QTC931               CTTAAGAGAGGCCAATTTGGCGTCGTACTTCGGAAAACCGATCAACTTTTAAAAGCTGCC
QTC1012              CT----------------------------------------------------------
QTC1389              ------------------------------------------------------------
TcPhosResExp_DLD     CTTAAGAGAG-CCAATTTGGCGTCGTACTTCG-AAAACCGATCA----------------
TcPhosSusUxp_DLD     CTTAAGAGAGGCCAATTTGGCGTCGTACTTCGGAAAACCGATCA----------------
TcPhosResUxp_DLD     CTTAAGAGAG-CCAATTTGGCGTCGTACTTCGGAAAACCGATCA----------------


QTC4                 ------------
TcPhosSusExp_DLD     ------------
QTC931               CATTTTTTTCCG
QTC1012              ------------
QTC1389              ------------
TcPhosResExp_DLD     ------------
TcPhosSusUxp_DLD     ------------
TcPhosResUxp_DLD     ------------
```
