# Supplementary material for: Genes related to mitochondrial functions are differentially expressed in phosphine-resistant and -susceptible Tribolium castaneum
Source: BMC Genomics. 2015 Nov 18;16:968. doi: 10.1186/s12864-015-2121-0 (PMC4650509; doi:10.1186/s12864-015-2121-0)
Supplement: Additional file 7: — Summary of SNPs/INDELs found in transcripts encoding DLD from phoshine-susceptible (A, unexposed; B, exposed) or -resistant (C, unexposed; D, exposed) T. castaneum adults, when transcripts were assembled to QTC4 ((gi|399108166|gb|AFP20530.1|) using SeqManNGen (DNAStar). (PDF 115 kb) [file 12864_2015_2121_MOESM8_ESM.pdf]

Additional File 6. Summary of SNPs/INDELs found in transcripts encoding DLD from phoshine-susceptible (A, unexposed; B, exposed) or -resistant (C, unexposed; D, exposed) *T. castaneum* adults, when transcripts were assembled to QTC4 ((gi|399108166|gb|AFP20530.1|) using SeqManNGen (DNAStar).

A.

Contig Pos	Ref Pos	Type	Ref Base	Called Base	Genotype	Impact	Homopolymer	SNP %	P not ref	Q call	Depth	A Cnt	C Cnt	G Cnt	T Cnt	Deletion
163	122	SNP	C	G	Homo. Variant			73.30%	100.00%	60	288	1	-	211	0	3
276	202	SNP	C	T|C	Hetero. Ref.			63.20%	100.00%	13.9	190	0	-	0	120	1
511	379	SNP	C	T	Homo. Variant			84.30%	100.00%	60	178	0	-	0	150	10
573	430	SNP	G	G|A	Hetero. Ref.			49.00%	100.00%	60	102	50	0	-	0	0
619	469	SNP	G	G|C	Hetero. Ref.			57.80%	100.00%	59.63	83	0	48	-	0	0
726	556	SNP	T	T|C	Hetero. Ref.			28.00%	96.80%	14.76	107	0	30	1	-	0
881	681	Indel	G	-	Homo. Variant			74.40%	100.00%	48.84	78	1	1	-	1	58
1097	850	SNP	A	G|A	Hetero. Ref.			60.70%	100.00%	60	122	-	0	74	0	0
1294	1006	SNP	G	A	Homo. Variant			72.50%	100.00%	26.81	40	29	0	-	0	1
1315	1027	SNP	A	G|A	Hetero. Ref.			36.00%	95.70%	13.48	50	-	0	18	0	0
1426	1109	Indel	G	-	Homo. Variant			69.60%	100.00%	60	260	5	0	-	0	181
1457	1131	Indel	G	G|-	Hetero. Ref.			39.60%	100.00%	57.96	240	0	0	-	0	95
1729	1318	SNP	C	T|C	Hetero. Ref.			61.50%	100.00%	52.59	205	0	-	0	126	1
1820	1384	SNP	C	T	Homo. Variant			97.00%	100.00%	60	230	0	-	0	223	2
1910	1444	SNP	T	T|A	Hetero. Ref.			16.60%	100.00%	60	511	85	0	0	-	3
1977	1486	Indel	C	C|-	Hetero. Ref.			45.30%	100.00%	60	492	1	-	1	0	223
1996	1499	Indel	G	G|-	Hetero. Ref.			43.40%	100.00%	60	442	0	3	-	0	192
2011	1511	Indel	-	G	Homo. Variant		Homopolymer	87.10%	100.00%	60	388	0	0	338	0	50
2042	1532	Indel	-	G	Homo. Variant		Homopolymer	95.40%	100.00%	60	174	0	1	166	0	7

B.

Contig Pos	Ref Pos	Type	Ref Base	Called Base	Genotype	Impact	Homopolymer	SNP %	P not ref	Q call	Depth	A Cnt	C Cnt	G Cnt	T Cnt	Deletion
165	122	SNP	C	G	Homo. Variant			73.10%	100.00%	60	316	0	-	231	0	2
274	202	SNP	C	T	Homo. Variant			84.60%	100.00%	60	246	0	-	0	208	1
514	379	SNP	C	T	Homo. Variant			85.00%	100.00%	60	240	0	-	0	204	12
576	430	SNP	G	G|A	Hetero. Ref.			71.60%	100.00%	18.67	95	68	0	-	0	0
620	469	SNP	G	C	Homo. Variant			80.20%	100.00%	34.24	86	0	69	-	0	0
901	681	Indel	G	-	Homo. Variant			77.70%	100.00%	60	148	0	1	-	0	115
1116	850	SNP	A	G|A	Hetero. Ref.			71.30%	100.00%	16.38	129	-	0	92	1	0
1318	1006	SNP	G	A	Homo. Variant			79.80%	100.00%	60	89	71	0	-	0	0
1453	1109	Indel	G	-	Homo. Variant			67.20%	100.00%	45.16	348	3	5	-	0	234
1486	1131	Indel	G	G|-	Hetero. Ref.			38.00%	100.00%	49.64	326	0	0	-	0	124
1764	1318	SNP	C	T|C	Hetero. Ref.			67.30%	100.00%	1.64	312	0	-	0	210	0
1853	1384	SNP	C	T	Homo. Variant			99.60%	100.00%	60	279	0	-	0	278	0
1949	1444	SNP	T	T|A	Hetero. Ref.			14.40%	100.00%	60	612	88	0	1	-	0
2017	1486	Indel	C	C|-	Hetero. Ref.			44.80%	100.00%	60	572	2	-	0	2	256
2038	1499	Indel	G	G|-	Hetero. Ref.			36.10%	100.00%	60	548	0	3	-	1	198
2053	1510	Indel	-	G|-	Hetero. Ref.		Homopolymer	73.60%	100.00%	60	478	1	0	352	0	125
2084	1532	Indel	-	G	Homo. Variant		Homopolymer	90.70%	100.00%	60	193	0	0	175	0	18

C.

Contig Pos	Ref Pos	Type	Ref Base	Called Base	Genotype	Impact	Homopolymer	SNP %	P not ref	Q call	Depth	A Cnt	C Cnt	G Cnt	T Cnt	Deletion
212	147	SNP	C	T|C	Hetero. Ref.			19.90%	100.00%	60	408	0	-	0	81	7
249	173	SNP	C	T|C	Hetero. Ref.			69.20%	100.00%	60	455	0	-	1	315	0
256	180	Indel	G	G|-	Hetero. Ref.			38.60%	100.00%	60	404	2	3	-	0	156
303	214	SNP	C	C|A	Hetero. Ref.			31.90%	95.10%	12.88	188	60	-	0	0	0
362	259	SNP	C	T|C	Hetero. Ref.			33.50%	99.70%	24.94	230	0	-	0	77	0
367	262	SNP	C	T|C	Hetero. Ref.			32.80%	97.80%	16.42	229	0	-	0	75	0
538	379	SNP	C	T|C	Hetero. Ref.			59.10%	100.00%	60	401	0	-	2	237	20
604	430	SNP	G	G|A	Hetero. Ref.			58.60%	100.00%	60	145	85	0	-	0	0
660	469	SNP	G	G|C	Hetero. Ref.			42.40%	100.00%	60	125	0	53	-	0	0
958	680	Indel	G	G|-	Hetero. Ref.			13.70%	100.00%	56.86	291	0	1	-	0	40
960	681	Indel	G	G|-	Hetero. Ref.			53.70%	100.00%	60	281	3	0	-	2	151
1176	850	SNP	A	G	Homo. Variant			94.90%	100.00%	60	295	-	0	280	0	0
1386	1006	SNP	G	A	Homo. Variant			93.70%	100.00%	60	95	89	0	-	0	1
1408	1027	SNP	A	G	Homo. Variant			100.00%	100.00%	60	15	-	0	15	0	0
1532	1109	Indel	G	G|-	Hetero. Ref.			69.60%	100.00%	60	483	3	8	-	1	336
1563	1131	Indel	G	G|-	Hetero. Ref.			39.80%	100.00%	60	467	0	0	-	0	186
1574	1139	Indel	T	T|-	Hetero. Ref.			19.20%	100.00%	60	437	0	0	0	-	84
1856	1318	SNP	C	T|C	Hetero. Ref.			25.40%	100.00%	60	564	1	-	0	143	0
1953	1384	SNP	C	T|C	Hetero. Ref.			55.40%	100.00%	60	437	0	-	0	242	0
2052	1444	SNP	T	T|A	Hetero. Ref.			30.30%	100.00%	60	967	293	1	0	-	4
2125	1486	Indel	C	C|-	Hetero. Ref.			43.90%	100.00%	60	883	2	-	5	4	388
2146	1499	Indel	G	G|-	Hetero. Ref.			39.00%	100.00%	60	772	0	0	-	0	301
2160	1510	Indel	-	G|-	Hetero. Ref.		Homopolymer	39.80%	100.00%	60	643	1	0	256	1	385
2162	1511	Indel	-	G|-	Hetero. Ref.		Homopolymer	48.10%	100.00%	60	624	0	0	300	2	322
2194	1532	Indel	-	G	Homo. Variant		Homopolymer	91.10%	100.00%	60	248	1	0	226	0	21

D.

Contig Pos	Ref Pos	Type	Ref Base	Called Base	Genotype	Impact	Homopolymer	SNP %	P not ref	Q call	Depth	A Cnt	C Cnt	G Cnt	T Cnt	Deletion
211	147	SNP	C	T|C	Hetero. Ref.			32.10%	100.00%	60	498	0	-	0	160	0
247	173	SNP	C	T|C	Hetero. Ref.			64.50%	100.00%	60	546	0	-	1	352	2
256	180	Indel	G	G|-	Hetero. Ref.			43.60%	100.00%	60	507	1	1	-	1	221
290	202	SNP	C	T|C	Hetero. Ref.			32.10%	100.00%	60	399	0	-	1	128	2
307	214	SNP	C	C|A	Hetero. Ref.			36.30%	100.00%	40.89	295	107	-	0	0	0
541	379	SNP	C	T|C	Hetero. Ref.			47.20%	100.00%	60	411	0	-	0	194	23
611	430	SNP	G	G|A	Hetero. Ref.			48.20%	100.00%	60	222	107	0	-	0	0
665	469	SNP	G	G|C	Hetero. Ref.			38.30%	100.00%	60	230	0	88	-	0	0
964	681	Indel	G	-	Homo. Variant			72.00%	100.00%	60	307	2	0	-	4	221
1202	850	SNP	A	G	Homo. Variant			96.10%	100.00%	60	331	-	0	318	0	0
1415	1006	SNP	G	A	Homo. Variant			97.00%	100.00%	60	100	97	0	-	0	0
1438	1027	SNP	A	G	Homo. Variant			95.50%	100.00%	60	22	-	0	21	0	1
1552	1109	Indel	G	G|-	Hetero. Ref.			68.30%	100.00%	60	710	2	5	-	1	485
1585	1131	Indel	G	G|-	Hetero. Ref.			41.20%	100.00%	60	699	0	0	-	1	288
1878	1318	SNP	C	T|C	Hetero. Ref.			22.50%	100.00%	60	677	0	-	1	152	0
1977	1384	SNP	C	T|C	Hetero. Ref.			57.20%	100.00%	60	594	1	-	2	340	1
2075	1444	SNP	T	T|A	Hetero. Ref.			31.70%	100.00%	60	1000	317	0	2	-	7
2149	1486	Indel	C	C|-	Hetero. Ref.			42.50%	100.00%	60	1000	3	-	4	3	425
2172	1499	Indel	G	G|-	Hetero. Ref.			43.80%	100.00%	60	994	0	2	-	0	435
2190	1511	Indel	-	G|-	Hetero. Ref.		Homopolymer	52.70%	100.00%	60	764	3	3	403	2	353
2191	1511	Indel	-	G|-	Hetero. Ref.		Homopolymer	36.30%	100.00%	60	764	0	0	277	1	486
2221	1532	Indel	-	G|-	Hetero. Ref.		Homopolymer	56.70%	100.00%	60	291	0	2	165	4	120
2222	1532	Indel	-	G|-	Hetero. Ref.		Homopolymer	36.40%	98.60%	18.56	291	0	0	106	4	181
