# Supplementary material for: Genes related to mitochondrial functions are differentially expressed in phosphine-resistant and -susceptible Tribolium castaneum
Source: BMC Genomics. 2015 Nov 18;16:968. doi: 10.1186/s12864-015-2121-0 (PMC4650509; doi:10.1186/s12864-015-2121-0)

Additional File 7. Treatment and sequencing strategy for *T. castaneum* adults (RFB). Abbreviations: RFBSusc, phosphine-susceptible laboratory colony; RFBPhosResBr, phosphine-resistant population from Brazil. Overall there were four treatments: RFBSusc not exposed to phosphine (blue); RFBPhosResBr not exposed to phosphine (orange); RFBSusc and RFBPhos-ResBr exposed to phosphine* (red). Each treatment was in triplicate; lower tier describes the samples that were extracted for mRNA used in the RNA-Seq experiments.

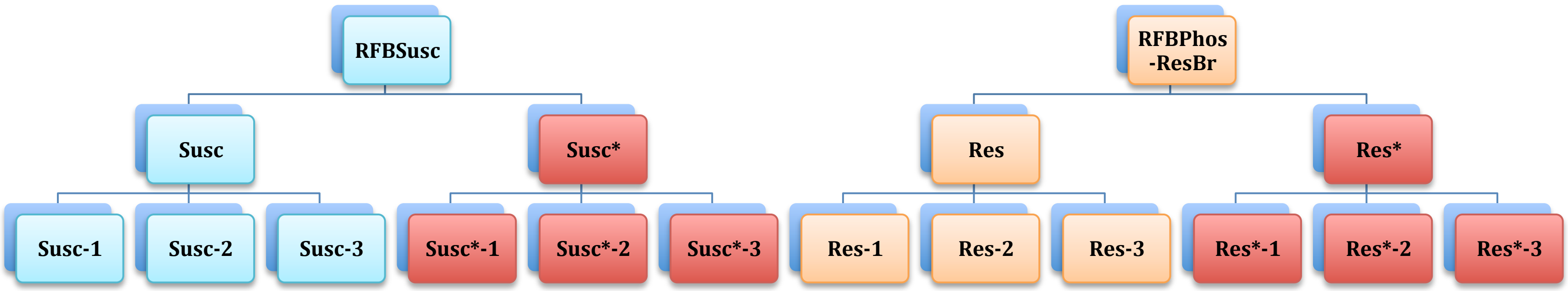

Supplement: Additional file 8: — Treatment and sequencing strategy for T. castaneum adults (RFB). Abbreviations: RFBSusc, phosphinesusceptible laboratory colony; RFBPhosResBr, phosphine-resistant population from Brazil. Overall there were four treatments: RFBSusc not exposed to phosphine (blue); RFBPhosResBr not exposed to phosphine (orange); RFBSusc and RFBPhos-ResBr exposed to phosphine* (red). Each treatment was in triplicate; lower tier describes the samples that were extracted for mRNA used in the RNA-Seq experiments. (PDF 278 kb) [file 12864_2015_2121_MOESM9_ESM.pdf]
