# Supplementary material for: Genes related to mitochondrial functions are differentially expressed in phosphine-resistant and -susceptible Tribolium castaneum
Source: BMC Genomics. 2015 Nov 18;16:968. doi: 10.1186/s12864-015-2121-0 (PMC4650509; doi:10.1186/s12864-015-2121-0)
Supplement: Additional file 9: — ArrayStar statistics on alignment of reads to Tcas3 (Total Reference bases - 32,060,158; Total Reference sequences - 18,428; Total Genome bases - 160,464,614). Parameters: mer length = 5; mer repeat threshold = fixed, 150; read assignment qualification - match 80 % of read or 20 bases. (PDF 60 kb) [file 12864_2015_2121_MOESM10_ESM.pdf]

Additional File 8. ArrayStar statistics on alignment of reads to Tcas3 (Total Reference bases - 32,060,158; Total Reference sequences - 18,428; Total Genome bases - 160,464,614).

Parameters: mer length = 5; mer repeat threshold = fixed, 150; read assignment qualification - match 80% of read or 20 bases.

	**ResExp 01**[1]	**ResExp 02**	**ResExp 03**	**Total ResExp**	**ResUxp 01**	**ResUxp 02**	**ResUxp 03**	**Total Resuxp**	**SusExp 01**	**SusExp 02**	**SusExp 03**	**Total SusExp**	**SusUxp 01**	**SusUxp 02**	**SusUxp 03**	**Total SusUxp**
Total reads	16824168	19815779	15212219	**51852166**	16631323	17586482	12766003	**46983808**	20514937	17285678	12590705	**50391320**	16648590	17854349	12893180	**47396119**
Total reads w/mer hits	14530416	16340426	12382515	**43253357**	13584157	14291077	10175677	**38050911**	17019317	14273357	10203766	**41496440**	13570165	13713733	10098926	**37382824**
Unique reads assigned	6703251	7060150	5797161	**19560562**	5523273	6086217	4085180	**15694670**	7480086	6519581	4740411	**18740078**	5655692	5403500	4253756	**15312948**
Repeated reads assigned	2387513	2721468	2122131	**7231112**	1955708	2301045	1530588	**5787341**	2697014	2441803	1688472	**6827289**	2028529	1983276	1744441	**5756246**
Reads rejected	7733404	10034161	7292927	**25060492**	9152342	9199220	7150235	**25501797**	10337837	8324294	6165822	**24827953**	8964369	10467573	6996083	**26428025**

[1]Key: ResExp = resistant/phosphine-exposed; ResUxp = resistant/unexposed; SusExp = susceptible/phosphine-exposed; SusUxp = susceptible/unexposed; 01,02,03 = biological replicate number
